# MoS_2_ Lubricate-Toughened MXene/ANF Composites for Multifunctional Electromagnetic Interference Shielding

**DOI:** 10.1007/s40820-024-01496-0

**Published:** 2024-10-11

**Authors:** Jiaen Wang, Wei Ming, Longfu Chen, Tianliang Song, Moxi Yele, Hao Zhang, Long Yang, Gegen Sarula, Benliang Liang, Luting Yan, Guangsheng Wang

**Affiliations:** 1 School of Physical Science and Engineering, Beijing Jiaotong University, Beijing, 100044 People’s Republic of China; 2 Key Laboratory of Bio-Inspired Smart Interfacial Science and Technology of Ministry of Education, School of Chemistry, Beihang University, Beijing, 100191 People’s Republic of China

**Keywords:** MXene–MoS_2_, Lubrication toughening, EMI shielding, Photothermal conversion, Electric heating performance

## Abstract

The design and fabrication of high toughness electromagnetic interference (EMI) shielding composite films with diminished reflection are an imperative task to solve electromagnetic pollution problem. Ternary MXene/ANF (aramid nanofibers)–MoS_2_ composite films with nacre-like layered structure here are fabricated after the introduction of MoS_2_ into binary MXene/ANF composite system. The introduction of MoS_2_ fulfills an impressive “kill three birds with one stone” improvement effect: lubrication toughening mechanical performance, reduction in secondary reflection pollution of electromagnetic wave, and improvement in the performance of photothermal conversion. After the introduction of MoS_2_ into binary MXene/ANF (mass ratio of 50:50), the strain to failure and tensile strength increase from 22.1 $\pm$ 1.7% and 105.7 $\pm$ 6.4 MPa and to 25.8 $\pm$ 0.7% and 167.3 $\pm$ 9.1 MPa, respectively. The toughness elevates from 13.0 $\pm$ 4.1 to 26.3 $\pm$ 0.8 MJ m^−3^ (~ 102.3%) simultaneously. And the reflection shielding effectiveness (SE_R_) of MXene/ANF (mass ratio of 50:50) decreases ~ 10.8%. EMI shielding effectiveness (EMI SE) elevates to 41.0 dB (8.2–12.4 GHz); After the introduction of MoS_2_ into binary MXene/ANF (mass ratio of 60:40), the strain to failure increases from 18.3 $\pm$ 1.9% to 28.1 $\pm$ 0.7% (~ 53.5%), the SE_R_ decreases ~ 22.2%, and the corresponding EMI SE is 43.9 dB. The MoS_2_ also leads to a more efficient photothermal conversion performance (~ 45 to ~ 55 °C). Additionally, MXene/ANF–MoS_2_ composite films exhibit excellent electric heating performance, quick temperature elevation (15 s), excellent cycle stability (2, 2.5, and 3 V), and long-term stability (2520 s). Combining with excellent mechanical performance with high MXene content, electric heating performance, and photothermal conversion performance, EMI shielding ternary MXene/ANF–MoS_2_ composite films could be applied in many industrial areas. This work broadens how to achieve a balance between mechanical properties and versatility of composites in the case of high-function fillers.

**Supplementary Information:**

The online version contains supplementary material available at 10.1007/s40820-024-01496-0.

## Highlights

- The introduction of MoS_2_ generates a “kill three birds with one stone” effect to the original binary MXene/ANF system: lubrication toughening mechanical performance; reduction in secondary reflection pollution of electromagnetic wave; and improvement in the performance of photothermal conversion.
- After the introduction of MoS_2_ into MXene/ANF (60:40), the strain and toughness were increased by 53.5% (from 18.3% to 28.1%) and 61.7% (from 8.9 to 14.5 MJ m^−3^), respectively. Fortunately, the SE_R_ decreases by 22.4%, and the photothermal conversion performance was increased by 22.2% from ~ 45 to ~ 55 °C.

**Supplementary Information:**

The online version contains supplementary material available at 10.1007/s40820-024-01496-0.

## Graphical abstract

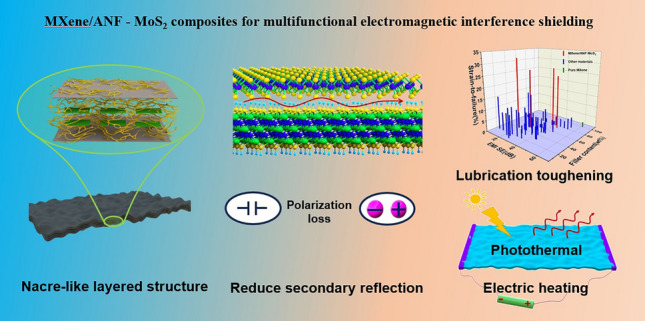

## Introduction

In recent years, the renewals of communication technology and flexible electronic devices lead to electromagnetic pollution risk [1–3]. Electromagnetic pollution has negative effects on other devices [4, 5] and human health [6–8]. The preparation of high toughness electromagnetic interference (EMI) shielding composite films becomes a major task to avoid the EM pollution problem. MXene has been utilized extensively as conductivity fillers in polymers-based EMI shielding composite films owing to its superior electrical conductivity [9–12], EMI shielding performance [13–15], mechanical performance [16, 17], electric heating performance [18–20], and photothermal conversion performance [21]. In addition, MXene could form interface interaction with soft polymers chains owing to the mass of functional groups on its surface [22–24].

In this background, substantial numbers of multilayer MXene/soft polymer chains composite films were prepared. Liu et al. [25] produced bacterial cellulose/MXene films via filtration method, the strain to failure was 17.3% with 20 wt% MXene, the corresponding EMI shielding effectiveness (EMI SE) was 18 dB, and the reflection shielding effectiveness (SE_R_) values are at a relatively high level. Peng et al. [26] fabricated reflection-dominated MXene/heterocyclic aramid nanocomposite films, the strain to failure was 16.2% and 3.5% with 20 and 60 wt% MXene, respectively, EMI SE value reaches 8.7 and 34.2 dB, respectively, and the reflectional coefficient values are larger than the absorption coefficients values. Nan et al. [27] adopted intermittent filtration method to realize the compound of MXene and aramid nanofibers (ANF); when the MXene content elevated from 20 to 60 wt%, the strain to failure decreased from 12.5 $\pm$ 0.8% to 5.3 $\pm$ 0.1%, but SE_R_ remained at a relatively high level. Teng et al. [28] produced reflection-dominated MXene/ANF EMI SE composite films, the strain to failure could reach 15.3 $\pm$ 1.0% with 20 wt% conductivity filler MXene, the corresponding EMI SE value was about 10 dB, the EMI shielding mechanism of the composite films is reflection dominated, and the strain to failure decreased to 5.4 $\pm$ 0.2% when the MXene content increased to 60 wt%.

It can be found that the high content conductivity filler will result in a descending tendency of mechanical performance. However, the high content of conductivity filler in EMI shielding composite materials is the decisive condition to fulfill superior EMI shielding performance. Therefore, under the condition of high content conductivity filler MXene, it is a challenge to prepare superior EMI shielding composite films with high toughness. On the other hand, the high electric conductivity of MXene also results in the high reflection of electromagnetic waves, which causes secondary reflection pollution [29]. Therefore, the fabrication of EMI shielding MXene-based composite films with excellent mechanical performance and diminished reflection simultaneously is a mainstream trend and a major challenge.

The layered structure of natural nacre provides inspiration for the preparation of MXene-based composite films. Natural nacre which possesses excellent mechanical performance has an alternately stacking multilayered structure which is composed of platelets and biopolymers; the platelets are embedded into biopolymer network [30, 31]. The platelets could concentrate the stress that transferred from biopolymer, and the rupture of biopolymers networks structure could absorb energy to fulfill the high toughness of natural nacre [32]. Inspired by the hierarchical structure of the natural nacre, abundant binary nacre-like MXene-based composite films were produced, such as MXene/ANF composite films [33] and MXene/MMT/SA films [34].

Commercial Kelva fiber could transform into aramid nanofibers (ANF). The ANF have a large specific area, high aspect ratio, and superior mechanical performance. After protonation process of ANF, the nanofibers could convert into interconnected networks which could enhance the mechanical performance of the composite films. Therefore, ANF have been compound with various functional fillers to fabricate various functional composite films with outstanding mechanical performance, for example, BN/ANF [35] (strain to failure up to 49.3%), NTS/ANF [36] (toughness up to 109 MJ m^−3^), BNNS/ANF–AgNWs@BNNS-BNNS/ANF [37] (tensile strength up to 245.9 MPa). MoS_2_ has been widely used in many areas owing to its multiple outstanding characteristics, such as lubrication toughening performance [38] and microwave absorption performance [39, 40]. Zhang et al. [41] prepared MXene/MoS_2_ microspheres; the interface polarization and the dielectric loss of MoS_2_ enhance electromagnetic wave attenuation. Wu et al. [42] compounded the MoS_2_ on the MXene, the MoS_2_/MXene exhibits a superior microwave absorption performance, and reflection loss (RL) value reaches − 60.2 dB at 16.6 GHz. MoS_2_ also possesses superior photothermal conversion performance. Luo et al. [43] compounded the MoS_2_ with quaternized chitosan (QCS) and cellulose nanofiber (CNF). The final MoS_2_@QCS/CNF composite paper exhibits superior photothermal conversion efficiency than the QCS/CNF composite paper. Herein, based on binary MXene/ANF composite films, ternary synergistic toughening MXene/ANF–MoS_2_ EMI shielding composite films are fabricated via vacuum-assisted filtration, self-assembly, and hot-pressing process. After the introduction of MoS_2_ nanosheets, MoS_2_ generates an exceptional positive “kill three birds with one stone” improvement effect in three aspects: lubrication toughening mechanical performance, diminished reflection EMI shielding performance, and more efficient photothermal conversion performance. Owing to the lubrication toughening effect of MoS_2_ nanosheets, the ternary MXene/ANF–MoS_2_ EMI shielding composite films exhibit an exceptional enhancement over the binary MXene/ANF composite films. After the introduction of MoS_2_ into binary MXene/ANF (mass ratio of 50:50) composite system, the strain to failure and tensile strength increase from 22.1 $\pm$ 1.7% and 105.7 $\pm$ 6.4 MPa to 25.8 $\pm$ 0.7% and 167.3 $\pm$ 9.1 MPa, respectively. The toughness elevates were increased by102.3% from 13.0 $\pm$ 4.1 to 26.3 $\pm$ 0.8 MJ m^−3^; After the introduction of MoS_2_ into the binary MXene/ANF (mass ratio of 60:40) composite films, the strain to failure was increased by 53.6% from 18.3 $\pm$ 1.9% to 28.1 $\pm$ 0.7%. The EMI SE of the MXene/ANF–MoS_2_ composite film is up to 43.9 dB in 8.2–12.4 GHz, while the SE_R_ significantly decreased by 22.2% (compared with MXene/ANF). The finding shows that the introduction of molybdenum disulfide nanosheets can not only ensure the composite excellent electromagnetic shielding performance, but also reduce the secondary pollution of electromagnetic wave. In addition, the ternary MXene/ANF–MoS_2_ composite films exhibit superior electric heating performance, quick temperature elevation (15 s), excellent cycle stability (2, 2.5, and 3 V), and long-term stability (2520 s). And the films possess a higher photothermal performance; the photothermal temperature of the composite film was increased by 22.2% from ~ 45 to ~ 55 °C. In summary, the integration of mechanical performance, EMI shielding performance, electric heating performance and photothermal conversion performance, and outstanding EMI shielding performance ensures the films could apply to many industrial areas.

## Material and Methods

### Materials

MoS_2_ dispersion was friendly furnished with other research institution. Aramid fiber (Kevlar 29). MAX (Ti_3_AlC_2_, Jilin 11 Technology Co., Ltd.). HCl (Modern Oriental Fine Chemistry, Beijing), LiF (Aladdin), KOH (Aladdin), DMSO (Aladdin).

### Preparation of Ternary MXene/ANF–MoS_2_ Films

Aramid nanofibers are fabricated by KOH deprotonation process. The mixed dispersion system which contains 5 g Kevlar 29 fibers, 5 g KOH, and 495 g DMSO is mechanical stirred persistently for one week, and the dark red viscous dispersion is ANF/DMSO. MXene is fabricated after the etching process of Ti_3_AlC_2_. The mixture which contains 1 g LiF, 20 mL 9 M HCl, and 1 g Ti_3_AlC_2_ is stirred persistently for one day. The cyclic centrifugal washing process of sediment is continued until pH of supernatant is more than 6. The ultrasonic treatment process of the sediment and the centrifugation process of dispersion continued 1 h, respectively. The resultant dark-green dispersion is the final MXene dispersion.

The MXene dispersion and MoS_2_ dispersion are added into ANF/DMSO dispersion in succession. After the process of stir, the homogeneous dispersion was filtered. Then, the obtained films are hot-pressed (100 °C, 10 min). ANF/MoS_2_ composite films are produced by above process without MXene. And the detailed different components additive amounts are shown in Table S1. In addition, binary MXene/ANF composite films are named AM, and ternary MXene/ANF–MoS_2_ composite films are named AMMO.

### Characterization

Transmission electron microscope (TEM) images were obtained with JEM-1400. Scanning electron microscope (SEM) images were performed by FE-SEM (JSM-7500F) and ESEM (Quanta 250FEG). The crystalline structures of materials were investigated by X-ray diffraction (XRD-6000). The chemical states were obtained by X-ray photoelectron spectroscope (XPS, Thermo escalab 250XI). The tensile stress–strain curves were obtained by tensile testing machine (JITAI100N, 1 mm min^−1^). The method for performing the EMI SE measurements is coaxial method, and the testing sample is a circular film with a diameter of 13 mm. S parameters were obtained by vector network analyzer (AV3618, CETC). EMI shielding effectiveness (EMI SE) SE_A_, SE_R_, and SE_T_ were calculated by the following formulas (SE_T_ ≥ 15 dB; multiple reflections SEM is ignored) [29, 33]:

1 $$T={\left|{S}_{12}\right|}^{2}={\left|{S}_{12}\right|}^{2}$$

2 $$R={\left|{S}_{11}\right|}^{2}={\left|{S}_{22}\right|}^{2}$$

3 $$A=1-T-R$$

4 $${\text{SE}}_{{\text{A}}} = - 10\log_{10} \left( {T - R} \right)$$

5 $${\text{SE}}_{{\text{R}}} = - 10\log_{10} \left( {1 - R} \right)$$

6 $${\text{SE}}_{{\text{T}}} = {\text{SE}}_{{\text{A}}} + {\text{SE}}_{{\text{R}}}$$

HIKMICRO (HM-TPK10-3AQF/W) recorded the variation in temperature. UTP3315TFL-II REGULATED DC POWER SUPPLY supplied different constant voltages in the electric heating performance testing. The illumination condition was provided by CEL-S500 in the photothermal conversion testing.

## Results and Discussion

### Preparation of Ternary MXene/ANF–MoS_2_ Composite Films

**Fig. 1:**
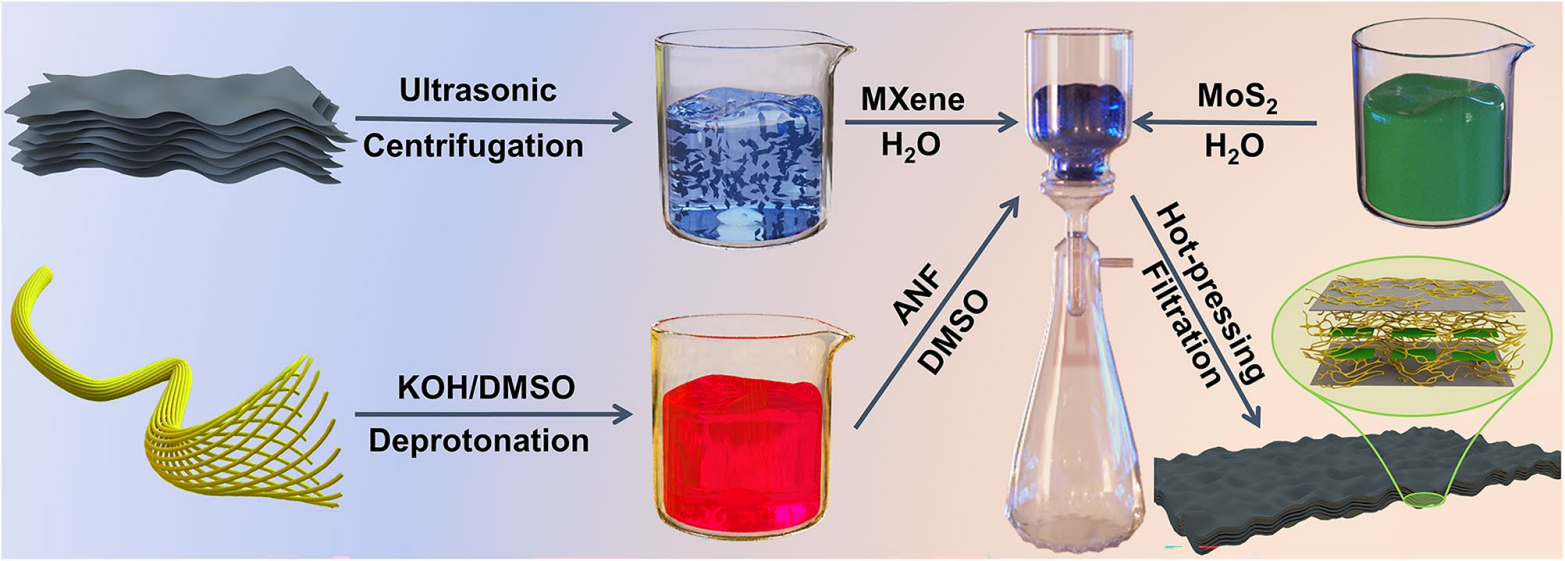
Fabrication process diagram of ternary MXene/ANF–MoS_2_ composite films

Figure 1 shows experimental preparation process diagram. Al atoms are etched in the mixture dispersion of LiF and HCl, and dense Ti_3_AlC_2_ (Fig. S1a) will convert into multilayer m-MXene (Fig. S1b). The dark-green MXene aqueous dispersion could be obtained after ultrasonic treatment process. Figure S1c shows the final MXene nanosheets. The hydrogen atoms of the Kevlar fibers molecule backbone are deprotonated in the mixture dispersion of KOH/DMSO [44]. The deprotonation process results in the decrease in the hydrogen bonds between polymers chains, which generates substantial numbers of negatively charged aramid nanofiber ANF chains. Figure S1d shows the TEM image of the final exfoliated ANF. The H_2_O in the MXene aqueous dispersion leads to the protonation of ANF [35, 45]. The interconnected ANF networks [36, 46], MXene nanosheets, and MoS_2_ nanosheets (Fig. S2) will stack and convert into the layered film structure after via vacuum-assisted filtration, self-assembly, and hot pressing. Figure S3a shows the cross-sectional SEM image; the structure is similar with layered natural nacre. The final digital picture of the flexible composite film is shown in Fig. S3b.

**Fig. 2:**
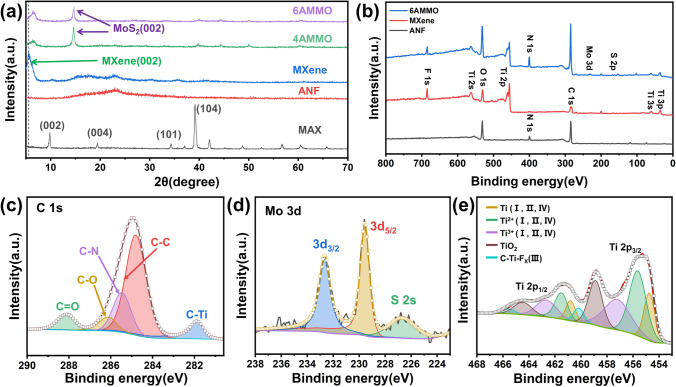
**a** XRD spectra of MXene, ANF, and ternary MXene/ANF–MoS_2_ composite films. **b** XPS spectra of MXene, ANF, and ternary MXene/ANF–MoS_2_ composite films. High-resolution XPS spectra of ternary MXene/ANF–MoS_2_ composite films: **c** C 1*s*; **d** Mo 3*d*; **e** Ti 2*p*

Figure 2a shows the XRD spectra of the MAX, MXene, ANF [33], and ternary MXene/ANF–MoS_2_ composite films. The characteristic peak (002) has an obvious shift to a large angle, which demonstrates the successful introduction of ANF and MoS_2_ into MXene. According to the XRD spectra of ANF/MoS_2_ and ANF/MXene–MoS_2_ (Fig. S4), it can be found the MoS_2_ is 2H crystal structure that has excellent lubrication effect [38]. Figures 2b and S5 show the XPS spectra of MXene, ANF [33], AMMO composite films, and MoS_2_. The N, Mo, and Ti elements peaks appear in the final ternary MXene/ANF–MoS_2_ spectrum. The high-resolution spectra for C 1*s* are shown in Fig. 2c, and the existence of C-Ti peak illustrates the incorporation of MXene. The high-resolution XPS spectra of Mo 3*d* and Ti 2*p* [23] are shown in Fig. 2d, e, which illustrate the successful incorporation of MXene, ANF, and MoS_2_. The fracture cross-sectional energy-dispersive spectrometry (EDS) mapping images are shown in Fig. S6. The distribution of the elements Ti, Mo, and S also demonstrates the successful incorporation of ANF, MXene, and MoS_2_.

### Mechanical Performance

**Fig. 3:**
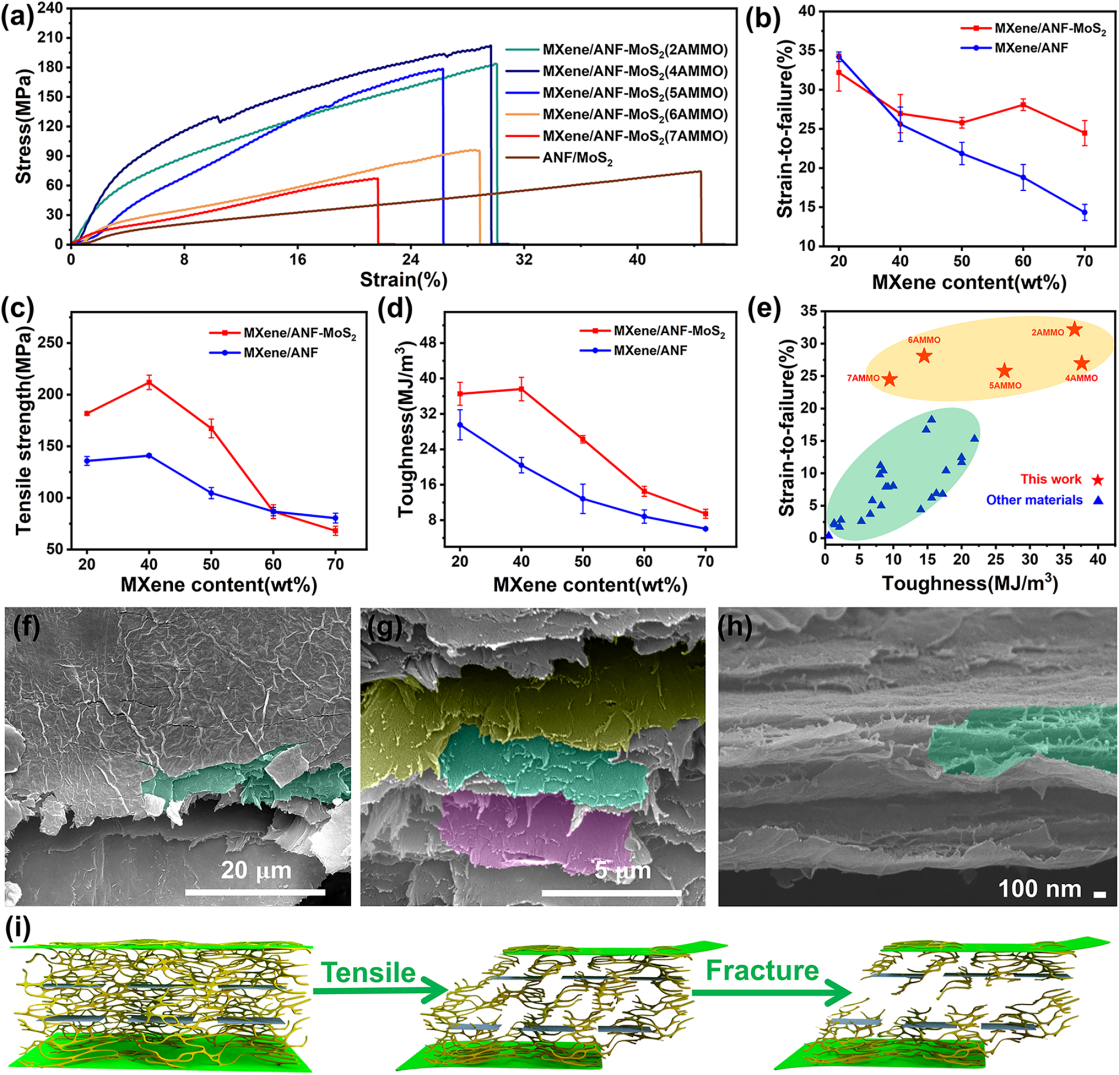
**a** Tensile stress–strain curves of ternary MXene/ANF–MoS_2_ composite films. The mechanical performance comparison between binary MXene/ANF and ternary MXene/ANF–MoS_2_ composite films: **b** strain to failure; **c** tensile strength; **d** toughness. **e** Mechanical performance comparison between ternary MXene/ANF–MoS_2_ and other composite films. **f** Fracture surface SEM image of 5AMMO. **g** Enlarged fracture surface image of 5AMMO. **h** Fracture cross-sectional SEM image of 6AMMO. **i** Fracture mechanism diagram of ternary MXene/ANF–MoS_2_ composite films

Based on binary MXene/ANF(AM) films, the ternary AMMO films exhibit an exceptional mechanical performance after the introduction of MoS_2_. The mechanical performance test results are shown in Figs. 3a and S7, S8. The ternary AMMO films all exhibit ultralong strain to failure and high tensile strength with various components content. Figure 3b-d shows the mechanical performance comparison between binary AM [33] and ternary AMMO films. For the binary MXene/ANF system with 20 and 40 wt% MXene, after the introduction of MoS_2_, the tensile strength obviously improves from 136.5 $\pm$ 5.1 and 140.9 $\pm$ 1.8 MPa to 181.8 $\pm$ 1.4 and 211.9 $\pm$ 7.0 MPa, respectively, and the strain to failure still maintains an extremely ultrahigh level simultaneously. After the introduction of MoS_2_ into MXene/ANF composite system with 60 and 70 wt% MXene, the strain to failure remarkably improves from 18.3 $\pm$ 1.9% and 14.4 $\pm$ 1.3% to 28.1 $\pm$ 0.7% and 24.5 $\pm$ 1.6%, respectively; at the same time, the toughness is 14.5 $\pm$ 1.1 and 9.4 $\pm$ 1.0 MJ m^−3^, respectively. After the introduction of MoS_2_ into binary MXene/ANF (mass ratio of 50:50) composite system, both tensile strength and strain to failure exhibit obvious enhancement, the strain to failure and tensile strength increase from 22.1 $\pm$ 1.7% and 105.7 $\pm$ 6.4 MPa to 25.8 $\pm$ 0.7% and 167.3 $\pm$ 9.1 MPa, respectively, and the toughness was elevated by 102.3% from 13.0 $\pm$ 4.1 to 26.3 $\pm$ 0.8 MJ m^−3^ simultaneously. The above results indicate that ternary MXene/ANF–MoS_2_ composite films still possess an ultrahigh toughness under high filler content condition, and the introduction of MoS_2_ generates a remarkable improvement to the mechanical performance of binary MXene/ANF system. The detailed mechanical performance statistics are shown in Table S2.

To rigorously evaluate the mechanical performance of the films, the mechanical property comparison statistic between ternary AMMO films and other materials is shown in Fig. 3e and Table S3. The ternary AMMO films with low filler content are located at the top right-hand corner, the result means the toughness, and the strain to failure all exceeds other composite materials. The ternary MXene/ANF–MoS_2_ composite films with higher filler content occupy the upper area, which means that the strain to failure still exceeds that of many other composite materials. In conclusion, ternary MXene/ANF–MoS_2_ composite films exhibit outstanding mechanical property.

Mechanical performance improvement caused after the introduction of MoS_2_ is investigated according to the SEM images. Figures 3f and S9 show the tensile fracture surface SEM images, it can be found that the layered structures generate an ultralong sliding distance which corresponds to the ultralong strain to failure, and the pulled-out layered structures form an obvious step structure (green region in Fig. 3f). Some microcracks which are parallel to fracture cross section exist on the film surface, which means that many tensile energy dissipation districts are present in the tensile deformation process. Figure 3g shows the enlarged image of the step structure, and the edges of the step structure are curved, which illustrates an improved interlayer interaction [23, 47]. When the stress is not sufficient to facilitate the sliding of the nanosheets, the adjacent nanosheets will slide owing to the stress transferred from the ANF networks. The continuously spread nanosheets sliding process results in the final pull-out step layered structure (colored regions in Fig. 3g) and crack deflection phenomenon, which correspond to the platelet “pull-out” mode and energy dissipation mechanism in the nacre [48].

Figures 3h and S10 show the fracture cross-sectional SEM images. AMMO films exhibit a nacre-like layered structure, and the layered structures are connected via the interconnected ANF networks (green region in Fig. 3h). And abundant pulled-out ANF nanofibers exist on the edge of the layered structure owing to the deformation and fracture of interconnected ANF networks. The EDS images of the pulled-out layered step structures are shown in Fig. S11; the elements Ti, Mo, and S have a uniform distribution on the layered step structure, which means the friction between MXene nanosheets and MoS_2_ nanosheets and the lubrication action of 2H MoS_2_ nanosheets all occur during the tensile deformation process [38]. At the same time, the MoS_2_ distribution in the cross section (Fig. S6) ensures that the lubrication toughening action could occur throughout the whole inner structure.

The fracture mechanism of the ternary AMMO films is proposed, and the tensile fracture mechanism diagram is shown in Fig. 3i. Owing to the hydrogen bonds between MXene and ANF which is weak, the sliding process of MXene nanosheets occurs in the initial tensile deformation process, the ANF networks deform, and the curved ANF nanofibers stretch preliminarily. The friction action between MXene nanosheets and MoS_2_ nanosheets results in MoS_2_ nanosheets sliding along the MXene nanosheets. At the same time, owing to the lubrication action of MoS_2_ nanosheets, the sliding of the MXene nanosheets could reach a greater extent. Under the condition of further tensile deformation, the nanofibers further stretch and break, and the above processes result in the formation of microcracks. After the deflected propagation process of the microcrack, more tensile energy is dissipated, resulting in the final fracture of the films.

### EMI Shielding Performance

**Fig. 4:**
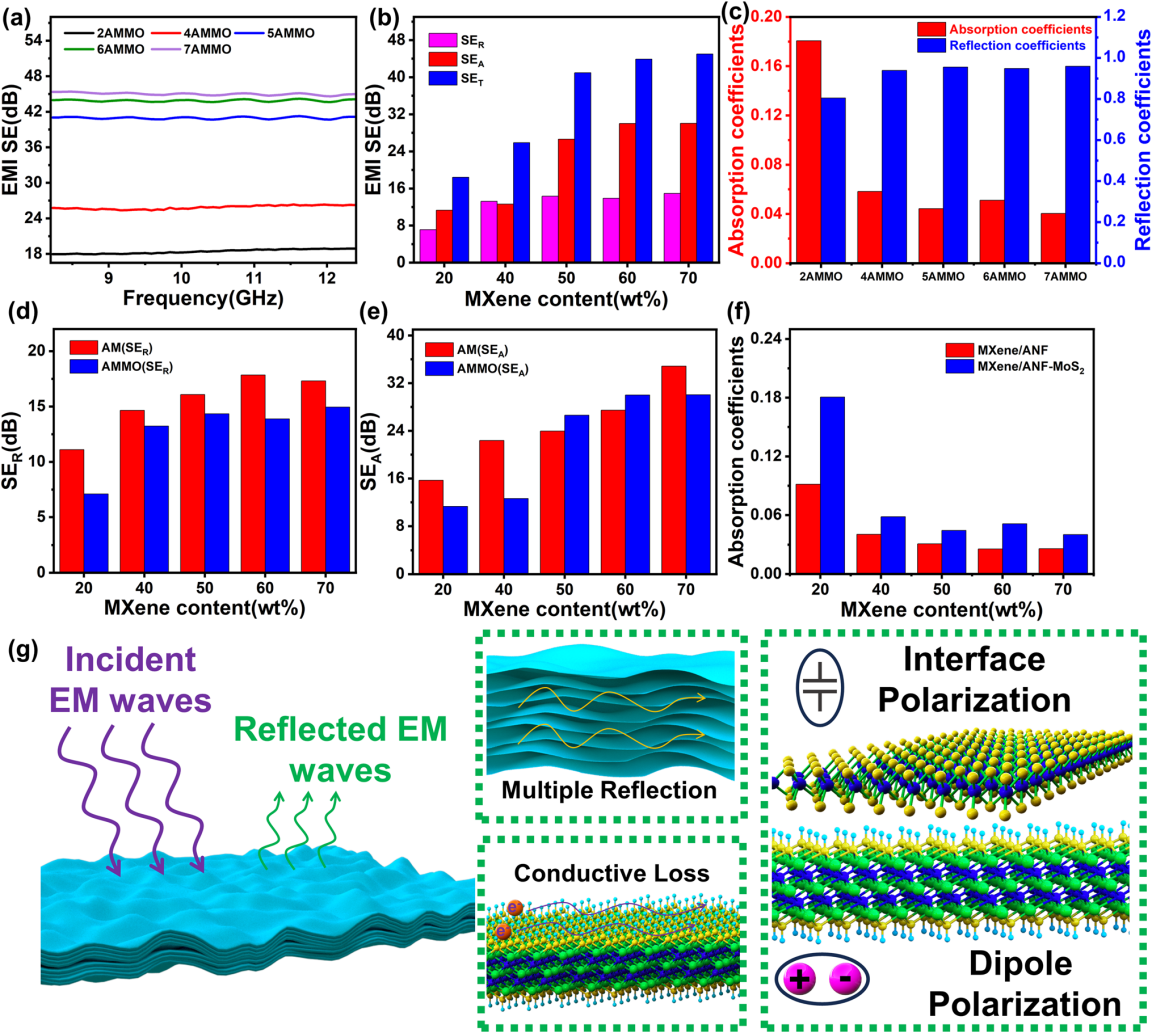
**a** EMI shielding effectiveness of ternary MXene/ANF–MoS_2_ composite films in X-band. **b** Average SE_R_, SE_A_, SE_T_ of ternary MXene/ANF–MoS_2_ composite films in X-band. **c** Average absorption coefficients A and reflection coefficients R of ternary MXene/ANF–MoS_2_ composite films in X-band. The comparison between binary MXene/ANF and ternary MXene/ANF–MoS_2_ composite films: **d** SE_R_; **e** SE_A_; **f** Absorption coefficients. **g** EMI shielding mechanism diagram of ternary MXene/ANF–MoS_2_ composite films

As shown in Fig. S12, AMMO composite films exhibit superior electric conductivity, and it is beneficial to the EMI shielding performance. Figure 4a, b and Table S4 exhibit detailed EMI shielding performance results. The EMI SE of 4AMMO and 6AMMO is 25.9 and 43.9 dB, respectively. The EMI SE values exceed the commercial electromagnetic shielding standard 20 dB [49, 50], which demonstrates the ternary MXene/ANF–MoS_2_ composite films could satisfy the practical application.

Figure 4c shows the average absorption coefficients A and reflection coefficients R of ternary MXene/ANF–MoS_2_ composite films in X-band. Owing to the presence of highly conductive MXene, the shielding mechanism of the composite is still mainly reflected, but the introduction of MoS_2_ nanosheets with wave-absorbing properties significantly reduces the emission of electromagnetic waves and the secondary pollution of electromagnetic waves (Fig. 4d-f) [33]. The SE_R_ values of film all exhibit an obvious decline; the SE_R_ of AMMO with 50 and 60 wt% MXene decreases by 10.8% and 22.2%, respectively, after the introduction of MoS_2_. Figure 4f illustrates the absorption coefficients exhibit an obvious enhancement after the introduction of the MoS_2_. The absorption coefficient A of the 2AMMO reaches 0.18. The introduction of MoS_2_ nanosheets which have applied in many MoS_2_-based microwave absorption materials leads to the rise of different interfaces. And the increased different interfaces lead to the charge accumulation which could enhance the interface polarization loss and interior multiple reflection of EM waves. The above results demonstrate the ternary MXene/ANF–MoS_2_ composite films have a diminished reflection EMI shielding mechanism, and the overall absorption EMI shielding effectiveness is improved to be more environment friendly to a certain extent via the introduction of MoS_2_. The above results indicate that MXene/ANF–MoS_2_ composite films also provide an avenue for fabricating diminished reflection EMI shielding composite films.

Figure S13 exhibits EMI SE result of ternary AMMO films in 1–18 GHz. The practical EMI shielding effect of the ternary AMMO films is checked. The electromagnetic radiation value decreases from 75.4 to 20.9 μW cm^−2^, and the warning of the electromagnetic radiation tester disappeared after the existence of the film (Fig. S14). The result corresponds to the multiband high efficiency EMI shielding performance. The ternary AMMO films possess obvious practical EMI shielding function and great practical application potential.

Figure 4g shows the EMI shielding mechanism diagram. Partial EM waves are reflected owing to the impedance mismatch [51] in EM waves incidence process. The other electromagnetic wave enters the inner structure of films. And the multiple reflection of electromagnetic wave takes place in the multiple layered structure. The migration and hopping of electrons on the surface of MXene will generate induced current which could effectively dissipate energy [52]. The surface functional groups of MXene result in dipole polarization loss of electromagnetic waves [53–55], which could further dissipate the electromagnetic waves energy. The charge accumulation at the interfaces between MXene nanosheets and MoS_2_ nanosheets results in interface polarization loss simultaneously [56]. And the increased interfaces also enhance the multiple reflection of EM waves. Based on the synergistic effect of the above factors, the ternary MXene/ANF–MoS_2_ composite films exhibit outstanding EMI SE performance.

### Thermal Performance

**Fig. 5:**
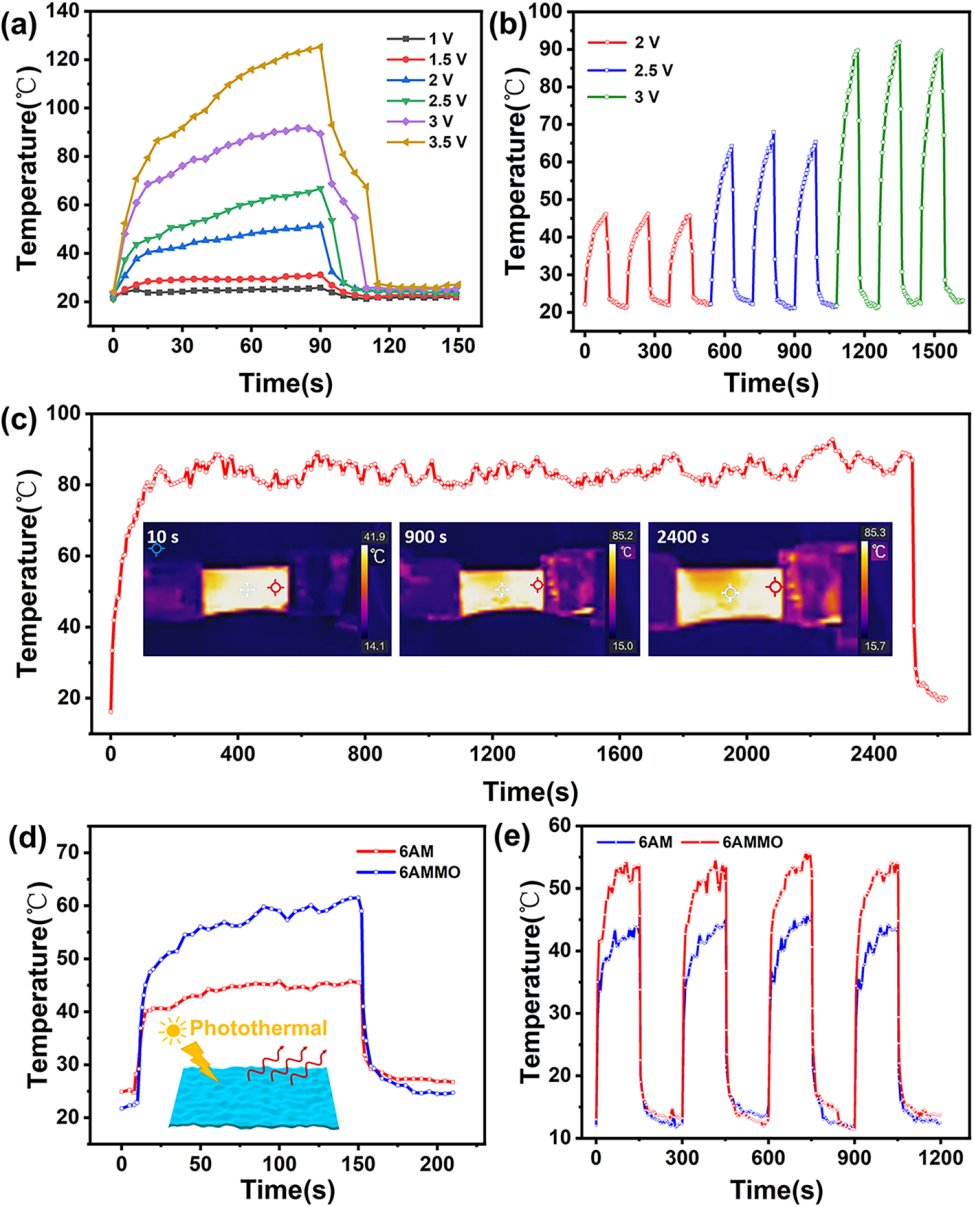
**a** Electric heating temperature variation curves under different voltages. **b** Repeated electric heating temperature variation curves. **c** Long-period electric heating temperature variation curves under different voltages. **d** Photothermal conversion temperature variation curves of binary MXene/ANF and ternary MXene/ANF–MoS_2_ composite films. **e** Repeated long-time photothermal conversion temperature variation curves of binary MXene/ANF and ternary MXene/ANF–MoS_2_ composite films

The ternary AMMO films exhibit superior electric heating performance because of the existence of MXene [57]. Herein, 6AMMO composite film is used as tested sample to measure the electric heating performance. Figure 5a shows the electric heating temperature alteration states. It can be found that the temperature could reach a relatively stable condition in a very short period (~ 15 s) and a higher temperature reached with the elevated applied voltages. The temperature could reach approximately 51 and 66 °C under 2 and 2.5 V, respectively.

The I–V curves are shown in Fig. S15a and the voltage and current also exhibit a linear relationship, which illustrates the MXene/ANF–MoS_2_ composite film maintains a steady resistance under various operation voltages. The electrical heating temperature and U^2^ (the square of electric heating voltages) exhibit a linear relationship, which also corresponds to the theoretical equation (Fig. S15b). Figure 5b shows the recycled electric heating temperature alteration states. The film could reach a stable recycle electric heating condition under various applied voltages. Figure S16 demonstrates the uniform distribution of the electric heating temperature of the AMMO composite films. Figure 5c shows the long period electric heating testing, and the temperature exhibits a relatively stable 80 °C within the 2520 s operation period. The above results demonstrate that the ternary AMMO films exhibit excellent electric heating performance, quick temperature elevation (15 s), excellent cycle stability (2, 2.5, and 3 V), and long-term stability (2520 s).

The photothermal conversion performance of binary AM and ternary AMMO films is tested in the room temperature with the same strength of illumination. The photothermal temperature variation curves are shown in Fig. 5d. The ternary AMMO film could reach a higher temperature level under a lower initial room temperature state, and the result demonstrates that the incorporation of MoS_2_ generates a remarkable improvement to the photothermal conversion performance. The higher photothermal conversion performance of ternary MXene/ANF–MoS_2_ could attribute to two aspects: compositional factor and structural factor. For the compositional factor, ternary MXene/ANF–MoS_2_ composite film is fabricated after the introduction of MoS_2_ into binary MXene/ANF composite system. Both the MXene and MoS_2_ nanosheets possess excellent photothermal performance, resulting in an increase in the photothermal conversion components in the ternary MXene/ANF–MoS_2_ composite film. As for the structure factor, the dense layered structure formed by MXene in the composite film forms an effective thermal conductivity network to transmit the heat generated by MXene and MoS_2_, which can achieve effective dissipation. The synergistic effect of compositional factor and structure factor leads to the ternary MXene/ANF–MoS_2_ composite films which exhibit better photothermal conversion performance.

Figure S17 shows the infrared thermal images, and the surfaces of films exhibit uniform temperature conditions. Figure 5e shows the cyclic long period photothermal conversion testing result of the films. During every photothermal conversion cyclic process, the composite films could reach a stable temperature in a short time and sustain a stable temperature. And the ternary 6AMMO film possesses a higher stable temperature simultaneously. The above results demonstrate that the ternary 6AMMO composite film possesses excellent photothermal conversion performance, high sensitivity, and long-term cyclic stability, and the introduction of MoS_2_ generates a remarkable improvement to the photothermal conversion performance.

### Comprehensive Property Comparison

**Fig. 6:**
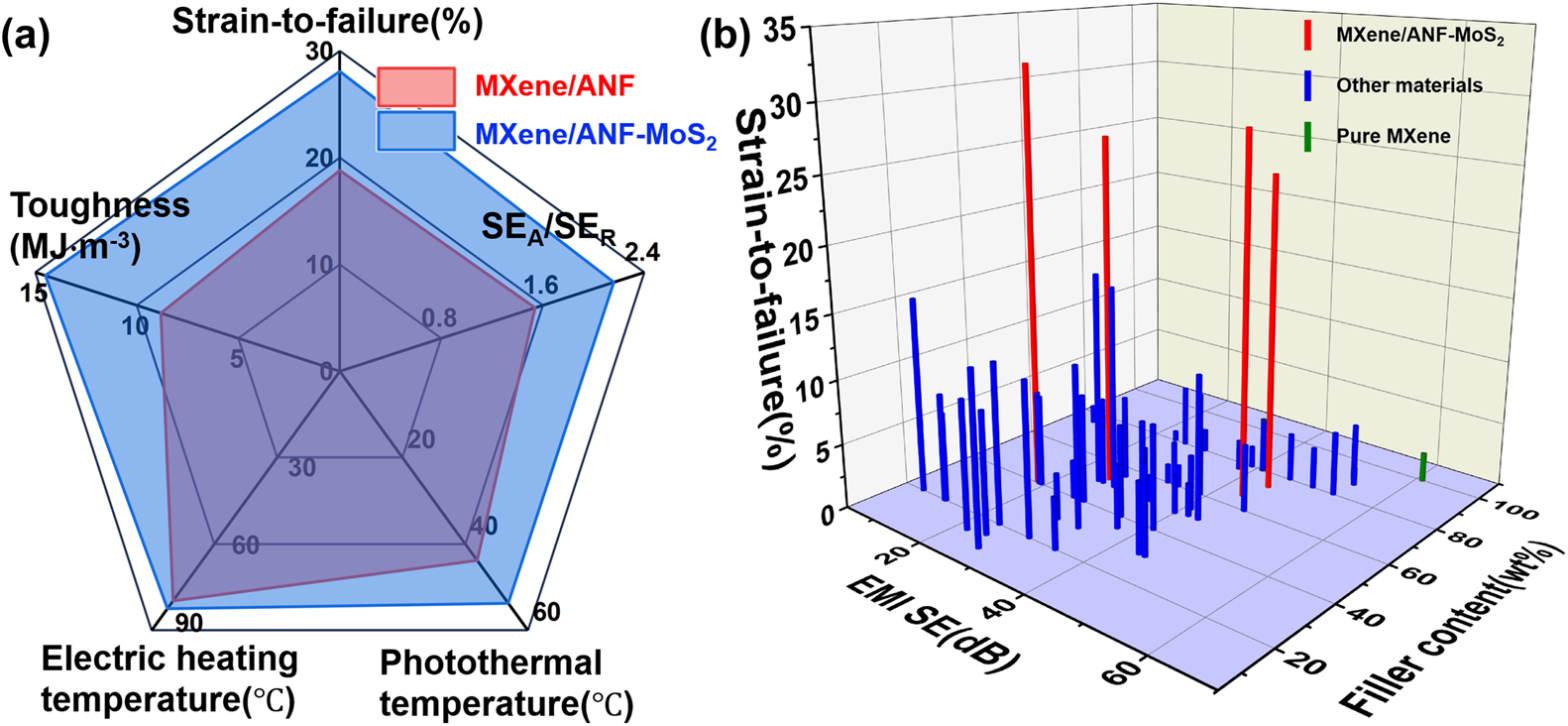
**a** Comparison between binary MXene/ANF composite films (6AM) and ternary MXene/ANF–MoS_2_ composite films (6AMMO). **b** Comparison between ternary MXene/ANF–MoS_2_ composite films and other MXene-based materials about strain to failure, EMI SE values, and filler content

To highlight the positive effects of the introduction of MoS_2_, Figs. 6a and S18, Tables S5 and S6 show the comparison about different aspects of MXene/ANF [33] and MXene/ANF–MoS_2_ composite systems. As shown in Fig. 6a, after the introduction of MoS_2_ into binary MXene/ANF (mass ratio of 60:40), the strain to failure increases from 18.3 $\pm$ 1.9% to 28.1 $\pm$ 0.7% (~ 53.5%), the SE_R_ decreases by 22.2%, and the corresponding EMI SE is 43.9 dB. The photothermal conversion performance of MXene/ANF composite system also exhibits obvious enhancement, from ~ 45 °C of 6AM to ~ 55 °C of 6AMMO. As shown in Fig. S18, after the introduction of MoS_2_ into binary MXene/ANF (mass ratio of 50:50) composite system, both strain to failure and tensile strength exhibit enhancement, and the toughness elevates from 13.0 $\pm$ 4.1 to 26.3 $\pm$ 0.8 MJ m^−3^ (~ 102.3%) simultaneously. The average EMI SE of corresponding ternary MXene/ANF–MoS_2_ composite system is 41.0 dB (8.2–12.4 GHz), and the SE_R_ of MXene/ANF composite system (mass ratio of 50:50) decreases by ~ 10.8%. The above comparisons demonstrate that the introduction of MoS_2_ causes a significantly improvement in three aspects of the binary MXene/ANF composite films [33]: EMI shielding performance, mechanical performance, and photothermal conversion performance. Figure 6b and Table S7 show the comprehensive performance comparison between ternary AMMO composite films and other MXene-based materials. The ternary MXene/ANF–MoS_2_ composite films exhibit a greatly advantage. Even though with high filler contents (total content proportion of MXene and MoS_2_) 65.9 and 73.4 wt%, the ternary MXene/ANF–MoS_2_ composite films still have ultralong strain to failure 28.1 $\pm$ 0.7% and 24.5 $\pm$ 1.6%, respectively. And the corresponding EMI SE reaches 43.9 and 45.0 dB, respectively. The above analysis results also illustrate that ternary MXene/ANF–MoS_2_ composite films could offer excellent mechanical property and EMI shielding performance with high content MXene simultaneously.

## Conclusion

The nacre-like layered ternary MXene/ANF–MoS_2_ composite films are produced via vacuum-assisted filtration, self-assembly, and hot-pressing process. After the introduction of MoS_2_ into binary MXene/ANF (mass ratio of 50:50), the strain to failure and tensile strength increase from 22.1 $\pm$ 1.7% and 105.7 $\pm$ 6.4 MPa and to 25.8 $\pm$ 0.7% and 167.3 $\pm$ 9.1 MPa, respectively, and the toughness elevates from 13.0 $\pm$ 4.1 to 26.3 $\pm$ 0.8 MJ m^−3^ (~ 102.3%) simultaneously. For the MXene/ANF (mass ratio of 60:40) composite system, the strain to failure increases from 18.3 $\pm$ 1.9% to 28.1 $\pm$ 0.7% after the introduction of MoS_2_, and the corresponding average EMI SE reaches 43.9 dB in X-band. At the same time, the SE_R_ value decreases by 22.2%. The MoS_2_ also leads to a more efficient photothermal conversion performance (~ 45 °C increased to ~ 55 °C). The above results indicate that the introduction of MoS_2_ achieves an effect of “kill three birds with one stone”: after the introduction of MoS_2_ into binary MXene/ANF composite system, the mechanical performance, EMI shielding performance, and photothermal conversion performance all exhibit a significant improvement. The ternary MXene/ANF–MoS_2_ composite films also possess excellent electric heating performance, quick temperature elevation (15 s), excellent cycle stability (2, 2.5, and 3 V), and long-term stability (2520 s). In conclusion, ternary MXene/ANF–MoS_2_ composite films offer a method for the preparation of EMI shielding composite films with ultrahigh toughness, and this work has great application potential in many industrial areas.

## Supplementary Information

Below is the link to the electronic supplementary material.

Supplementary file1 (DOCX 4099 KB)
