# Supplementary material for: MoS2 Lubricate-Toughened MXene/ANF Composites for Multifunctional Electromagnetic Interference Shielding
Source: Nanomicro Lett. 2024 Oct 11;17:36. doi: 10.1007/s40820-024-01496-0 (PMC11469983; doi:10.1007/s40820-024-01496-0)
Supplement: Supplementary file 1 — Supplementary file1 (DOCX 4099 KB) [file 40820_2024_1496_MOESM1_ESM.docx]

Supporting Information for

**MoS_2_ Lubricate-Toughened MXene/ANF Composites for Multifunctional Electromagnetic Interference Shielding**

Jiaen Wang^1, #^, Wei Ming^1, #^, Longfu Chen^1^, Tianliang Song^1^, Moxi Yele^1^, Hao Zhang^1^, Long Yang^1^, Gegen Sarula^1^, Benliang Liang^1,^ *, Luting Yan ^1,^ * and Guangsheng Wang ^2,^ *

^1^ School of Physical Science and Engineering, Beijing Jiaotong University, Beijing 100044, P. R. China

^2^ Key Laboratory of Bio-Inspired Smart Interfacial Science and Technology of Ministry of Education, School of Chemistry, Beihang University, Beijing 100191, P. R. China

^#^Jiaen Wang and Wei Ming contributed equally to this work.

*Corresponding authors. E-mail: [blliang@bjtu.edu.cn](mailto:blliang@bjtu.edu.cn) (Benliang Liang); [ltyan@bjtu.edu.cn](mailto:ltyan@bjtu.edu.cn) (Luting Yan); [wanggsh@buaa.edu.cn](mailto:wanggsh@buaa.edu.cn) (Guangsheng Wang)

**Supplementary Figures and Tables**


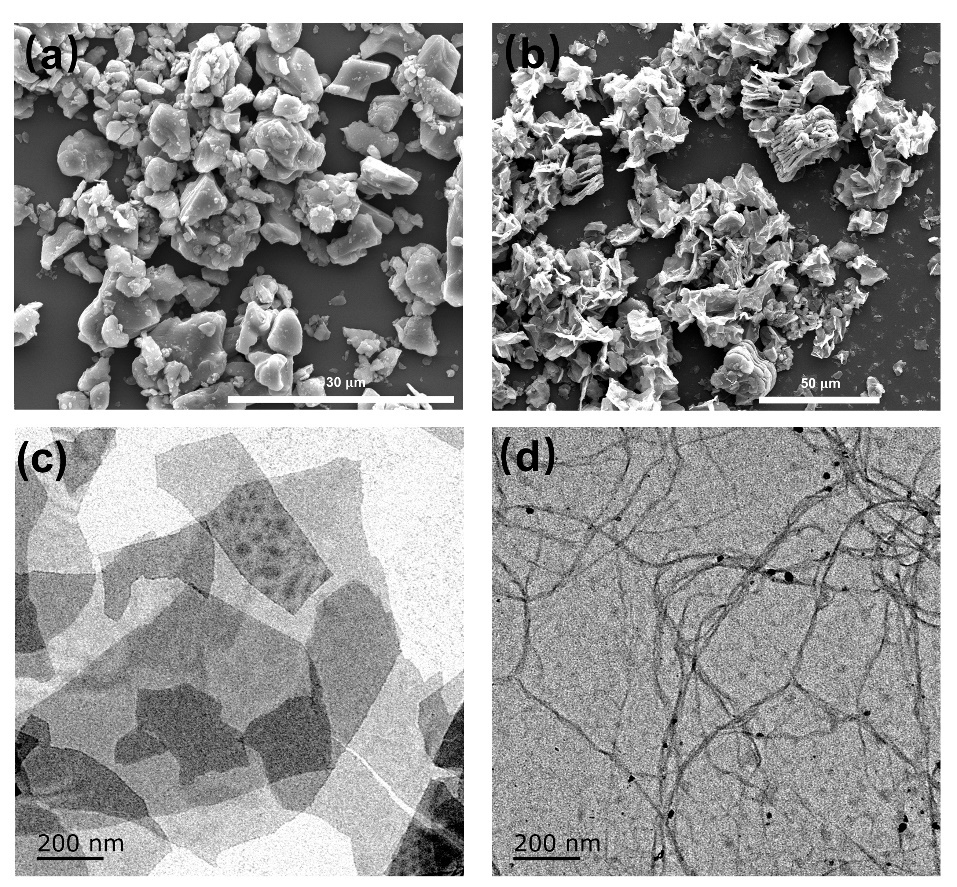


**Fig. S1** (**a**) SEM image of Ti_3_AlC_2_. (**b**) SEM image of m-MXene. (**c**) TEM image of MXene. (**d**) TEM image of ANF


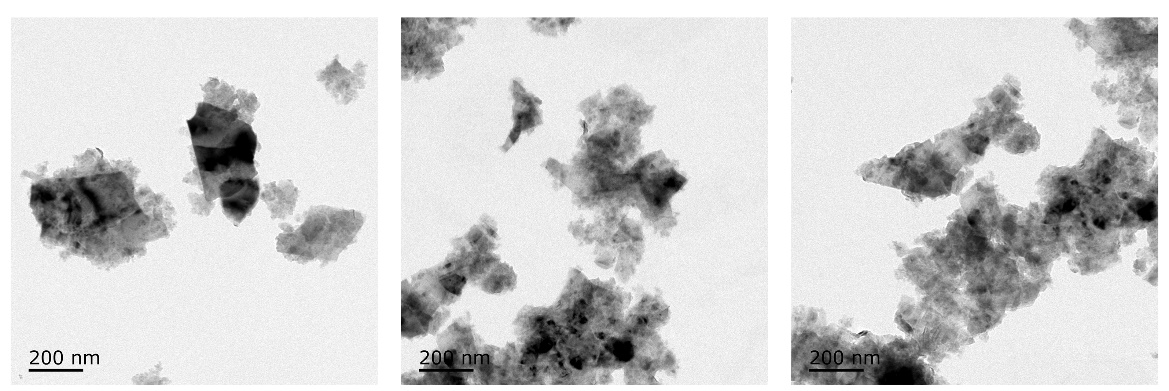


**Fig. S2** TEM images of MoS_2_


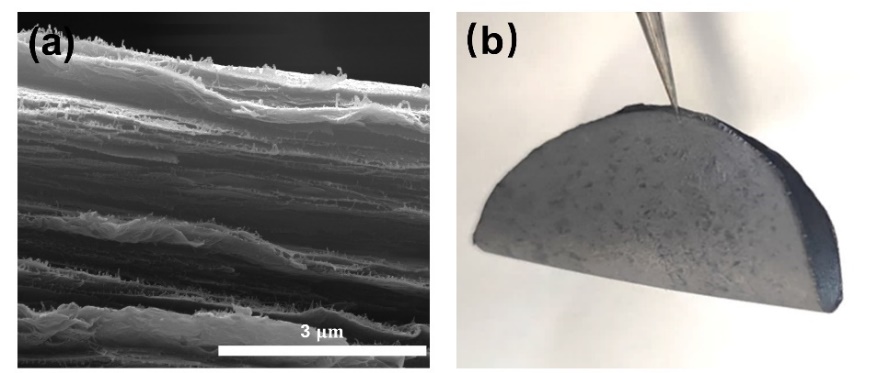


**Fig. S3** (**a**) Cross section SEM image of ternary MXene/ANF-MoS_2_ composite films. (**b**) Ternary MXene/ANF-MoS_2_ composite film digital picture


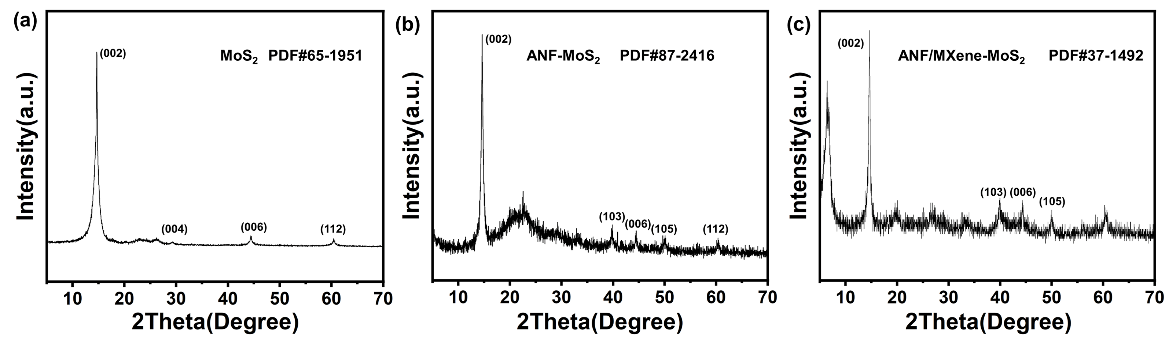


**Fig. S4** (**a**) XRD spectrum of MoS_2_. (**b**) XRD spectrum of ANF-MoS_2_ composite films. (**c**) XRD spectrum of ANF/MXene-MoS_2_


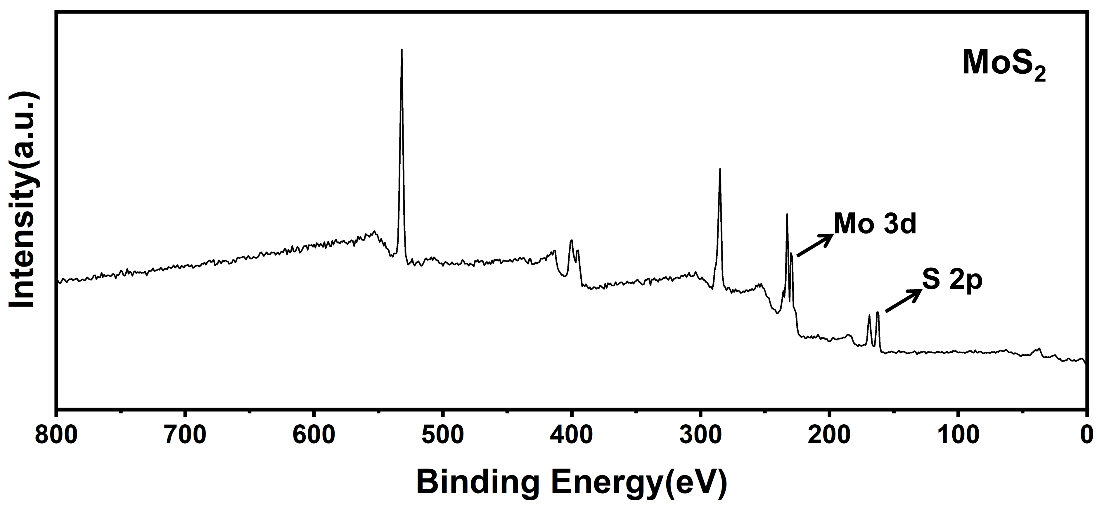


**Fig. S5** XPS spectrum of MoS_2_


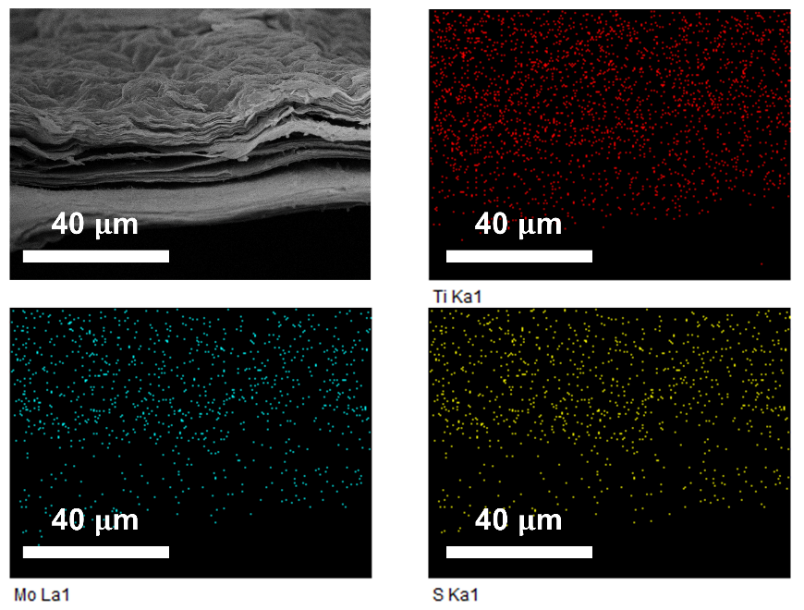


**Fig. S6** Fracture cross section EDS mapping images


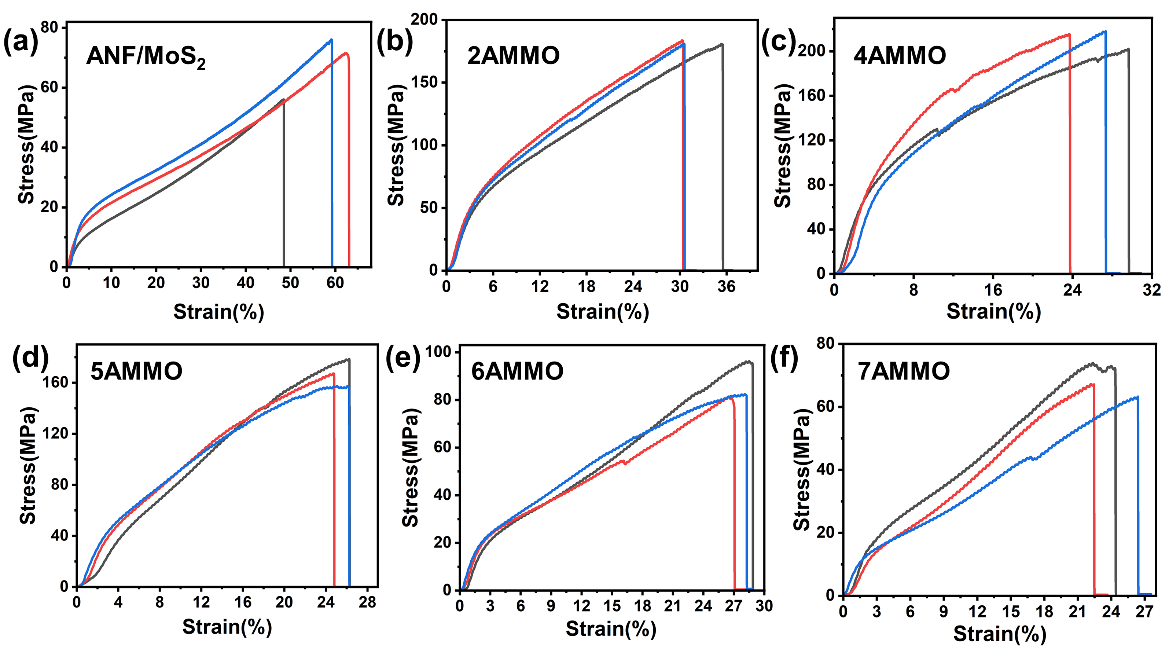


**Fig. S7** Tensile stress-strain curves of different composite films: (**a**) ANF/MoS_2_; (**b**) 2AMMO; (**c**) 4AMMO; (**d**) 5AMMO; (**e**) 6AMMO; (**f**) 7AMMO


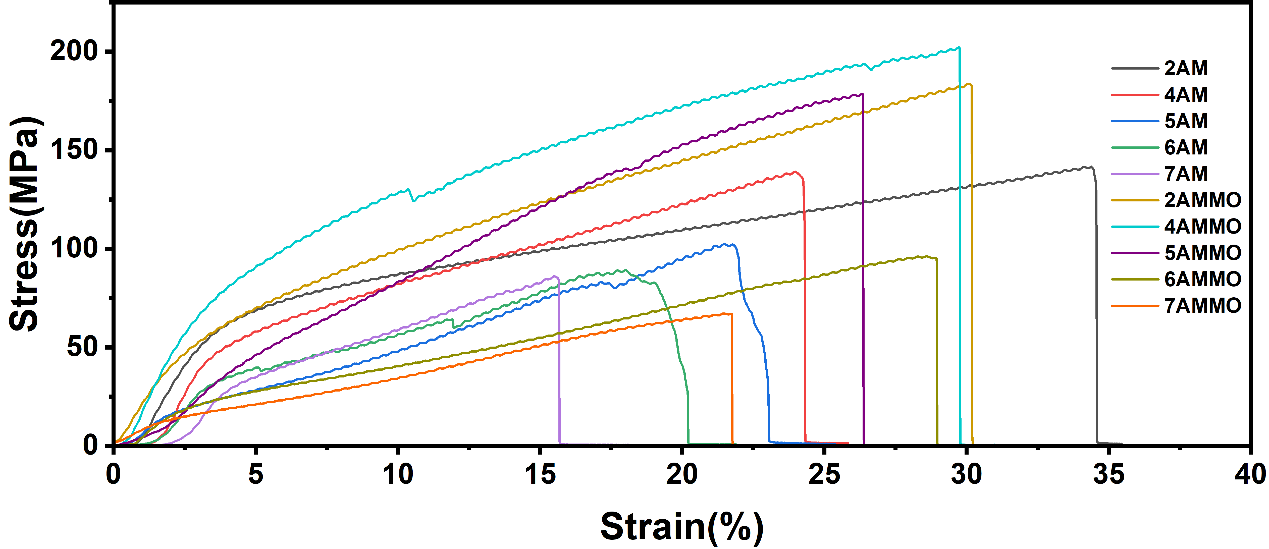


**Fig. S8** The tensile stress-strain curves of ternary MXene/ANF-MoS_2_ composite films and control samples: binary MXene/ANF composite films [S1]


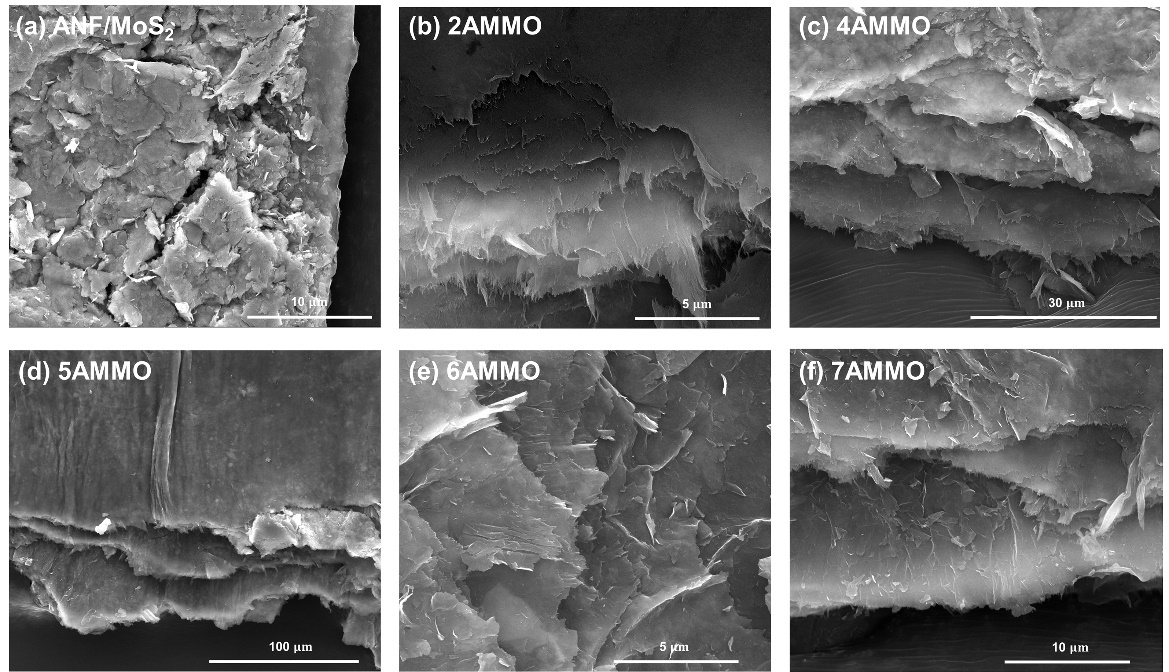


**Fig. S9** Fracture surface SEM images: (**a**) ANF/MoS_2_. (**b**) 2AMMO. (**c**) 4AMMO. (**d**) 5AMMO. (**e**) 6AMMO. (**f**) 7AMMO


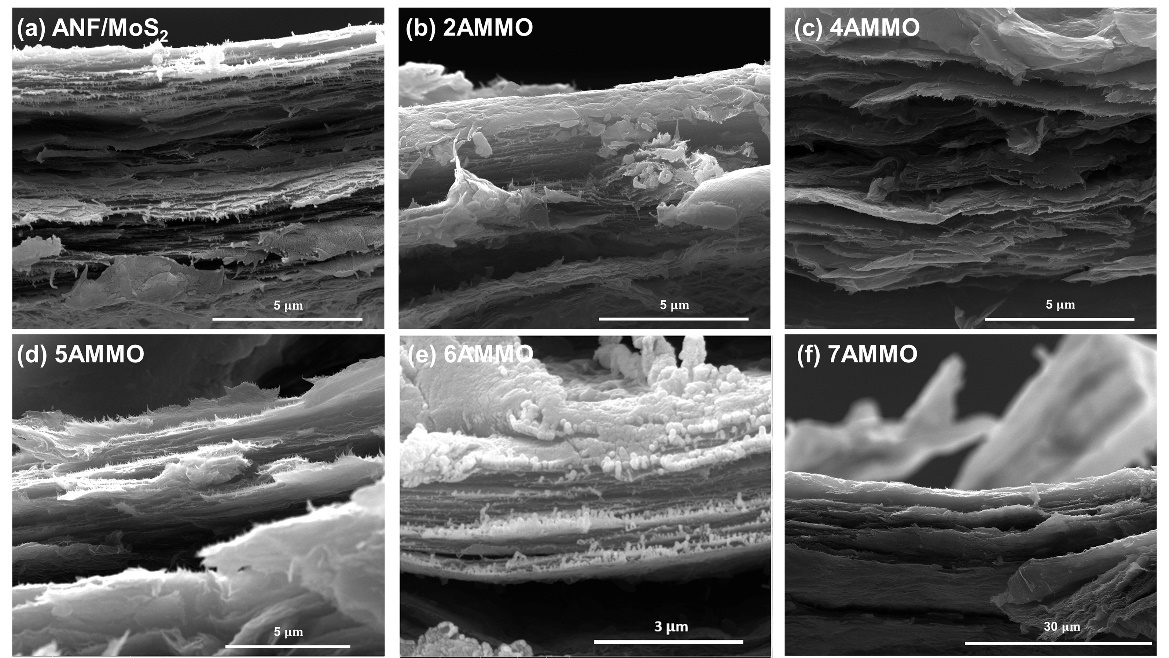


**Fig. S10** Fracture cross-section SEM images: (**a**) ANF/MoS_2_. (**b**) 2AMMO. (**c**) 4AMMO. (**d**) 5AMMO. (**e**) 6AMMO. (**f**) 7AMMO


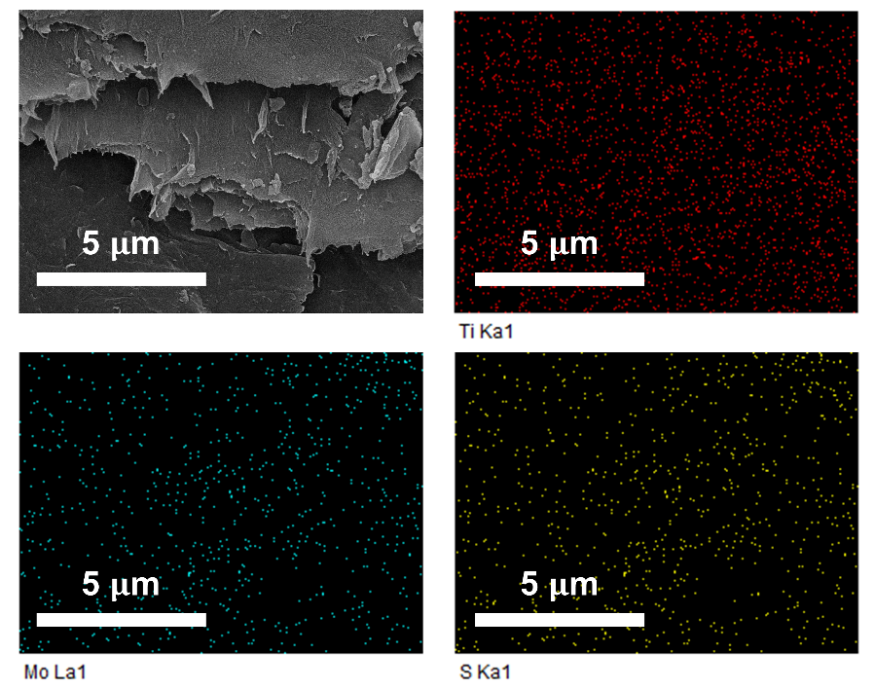


**Fig. S11** Fracture surface EDS mapping images


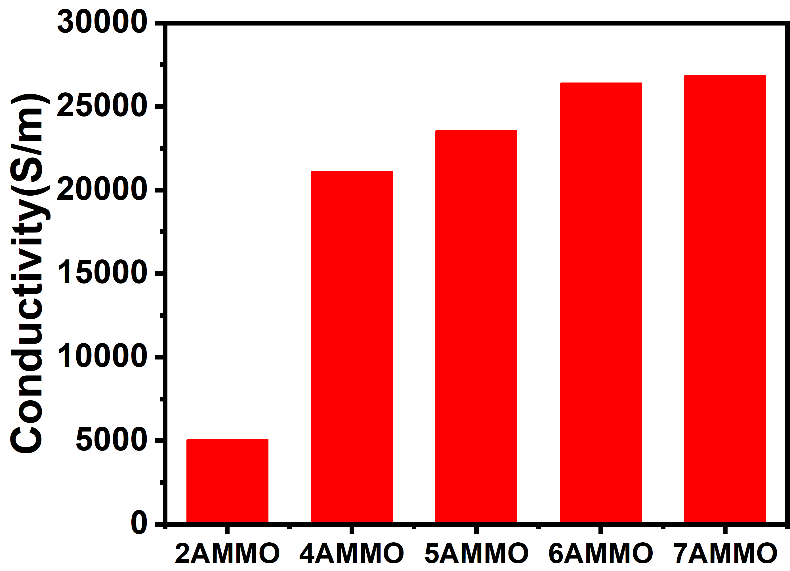


**Fig. S12** Electrical conductivity of AMMO composite films


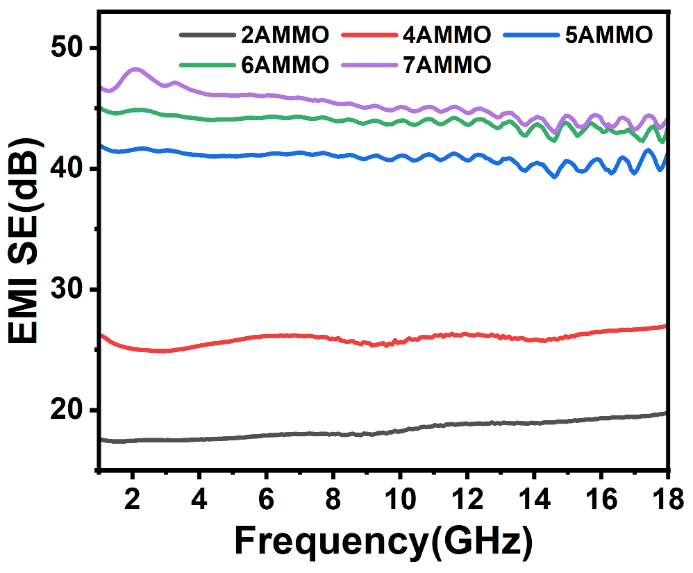


**Fig. S13** EMI shielding effectiveness of ternary MXene/ANF-MoS_2_ composite films in 1-18 GHz


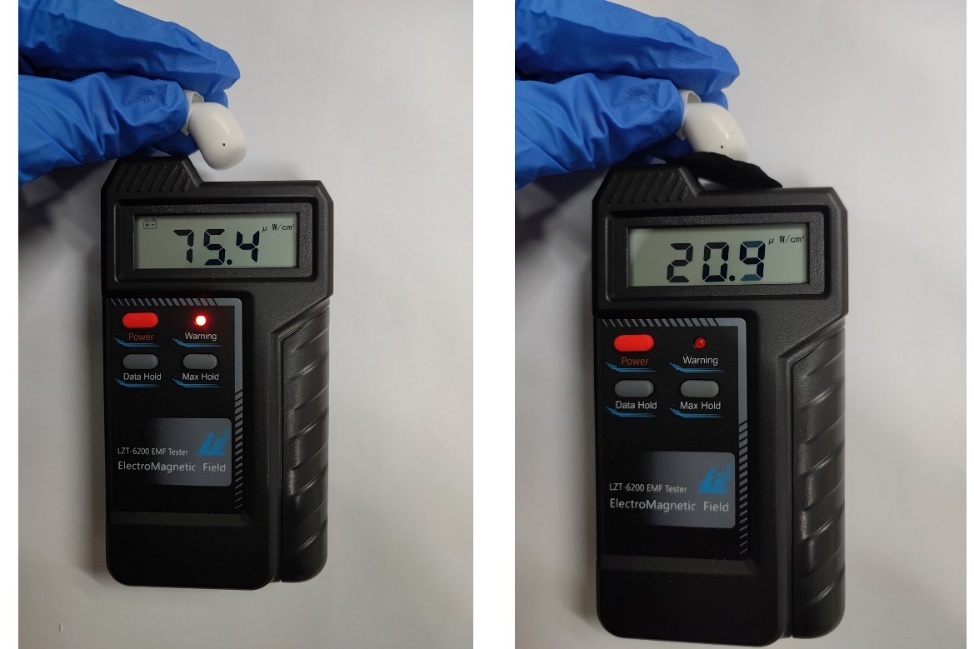


**Fig. S14** EMI shielding result of a Bluetooth headset


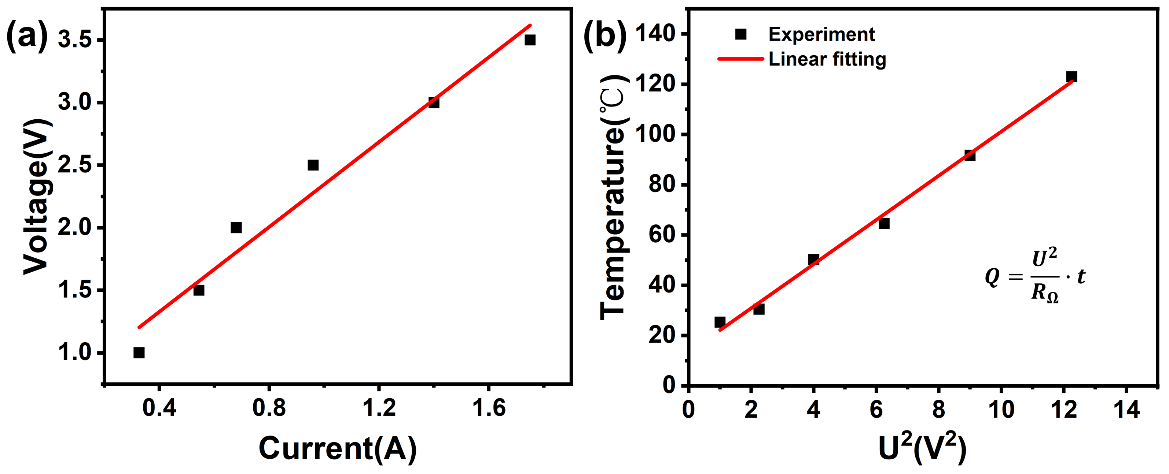


**Fig. S15** (**a**) I-V curve of ternary MXene/ANF-MoS_2_ composite films. (**b**) The linear fitting of temperature and U^2^


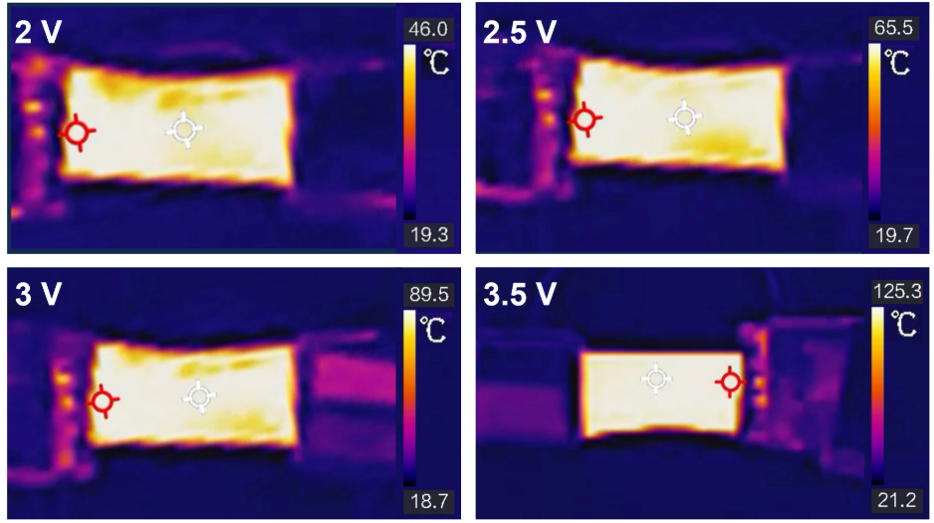


**Fig. S16** Infrared thermal images of ternary MXene/ANF-MoS_2_ composite films under different electric heating voltages


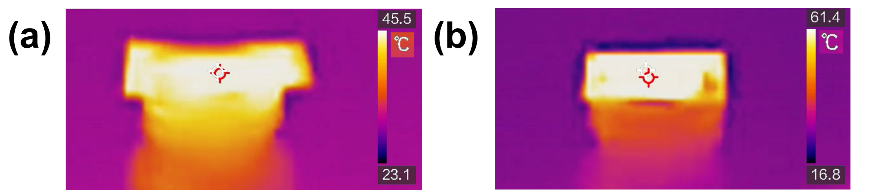


**Fig. S17** Infrared thermal images in photothermal conversion: (**a**) binary MXene/ANF composite films; (**b**) ternary MXene/ANF-MoS_2_ composite films


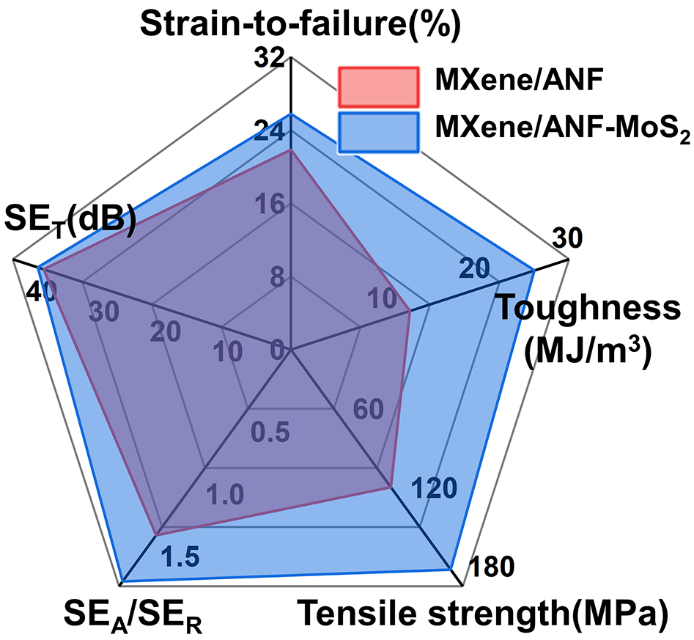


**Fig. S18** The comparison between binary 5AM and ternary 5AMMO

**Table S1** The detailed additive amounts of different components in the experimental preparation process

|  | **MXene：ANF** | **MXene**  **(0.29 wt%)** | **ANF**  **(1 wt%)** | **MoS_2_**  **(0.55 wt%)** | **Filler content** |
| --- | --- | --- | --- | --- | --- |
| ANF/MoS_2_ |  |  | 1.8 g | 1.4025 g | 0.3 |
| 2AMMO | 2:8 | 1.55 g | 1.8 g | 1.4025 g | 0.4041 |
| 4AMMO | 4:6 | 4.14 g | 1.8 g | 1.4025 g | 0.5228 |
| 5AMMO | 5:5 | 6.21 g | 1.8 g | 1.4025 g | 0.5883 |
| 6AMMO | 6:4 | 9.31 g | 1.8 g | 1.4025 g | 0.6585 |
| 7AMMO | 7:3 | 14.48 g | 1.8 g | 1.4025 g | 0.7341 |

**Table S2** Mechanical performance of MXene/ANF and MXene/ANF-MoS_2_ composite films

|  | **Tensile Strength (MPa)** | **Strain-to-failure (%)** | **Toughness (MJ∙m^-3^)** |
| --- | --- | --- | --- |
| ANF | 60.0 $\pm$ 2.9 | 44.0 $\pm$ 3.7 | 16.8 $\pm$ 2.8 |
| 2AM | 136.5 $\pm$ 5.1 | 34.0 $\pm$ 0.7 | 29.7 $\pm$ 4.1 |
| 4AM | 140.9 $\pm$ 1.8 | 26.3 $\pm$ 2.5 | 20.3 $\pm$ 2.1 |
| 5AM | 105.7 $\pm$ 6.4 | 22.1 $\pm$ 1.7 | 13.0 $\pm$ 4.1 |
| 6AM | 85.5 $\pm$ 4.4 | 18.3 $\pm$ 1.9 | 8.8 $\pm$ 1.8 |
| 7AM | 82.1 $\pm$ 5.1 | 14.4 $\pm$ 1.7 | 6.1 $\pm$ 0.3 |
| ANF/MoS_2_ | 67.9$\pm$8.6 | 57.0$\pm$6.2 | 21.4$\pm$5.0 |
| 2AMMO | 181.8$\pm$1.4 | 32.2$\pm$2.4 | 36.5$\pm$2.6 |
| 4AMMO | 211.9$\pm$7.0 | 27.0$\pm$2.4 | 37.6$\pm$2.6 |
| 5AMMO | 167.3$\pm$9.1 | 25.8$\pm$0.7 | 26.3$\pm$0.8 |
| 6AMMO | 86.7$\pm$6.8 | 28.1$\pm$0.7 | 14.5$\pm$1.1 |
| 7AMMO | 68.1$\pm$4.4 | 24.5$\pm$1.6 | 9.4$\pm$1.0 |

**Table S3** The comparison of toughness and strain-to-failure between ternary MXene/ANF-MoS_2_ composite films and other works

| **Materials** | **Strain-to-failure (%)** | **Toughness (MJ**$\boldsymbol{\cdot}$**m^-3^)** | **References** |
| --- | --- | --- | --- |
| MMT/PVA-GA 70/30 | 0.33 | 0.5 | [S2] |
| MTM/PVA 70/30 | 1.7 | 2.1 | [S3] |
| MTM/CS 65/35 | 2.3 | 1.3 | [S4] |
| MTM/PDDA 70/30 | 2.1 | 1.4 | [S5] |
| MMT/CMC 20/80 | 2.6 | ~5.3 | [S6] |
| RGO/CS 95/5 | 10.4 | 17.7 | [S7] |
| RGO/MoS2-TPU 86/14 | 5.8 | 6.9 | [S8] |
| RGO/CS-Cu^+^ 91/9 | 4.4 | 14 | [S9] |
| RGO/CMC-Mn^2+^ 87/13 | 3.7 | 6.6 | [S10] |
| RGO/PVA 58/42 | 10.4 | 8.5 | [S11] |
| GO/Al_2_O_3_-CMC 8/92 | 5 | 8.2 | [S12] |
| GO/PAA 95/5 | 7.9 | 8.9 | [S13] |
| MTM/NFC 50/50 | 2.8 | 2.3 | [S14, S15] |
| MXene/NFC 50/50 | 16.7 | 14.8 | [S16] |
| Aminoclay/TOCN 33/67 | 11.7 | 20 | [S17] |
| RGO/TOCN 95/5 | 6.2 | 15.6 | [S18] |
| RGO/DWNT 95/5 | 7.9 | 9.2 | [S19] |
| Alumina/CMC/Cu^2+^  13.3/20 | ~6.8 | 17.2 | [S20] |
| MXene/BN-PBO 25/75 | ~9.8 | ~8 | [S21] |
| MXene/ANF 20/80 | 15.3 | 21.87 | [S22] |
| MXene/ANF 20/80 | 12.45 | 19.97 | [S23] |
| MXene/  heterocyclic aramid  50/50 | 6.9 | 16.29 | [S24] |
| CuNW/  MXene/ANF  60/40 | 18.25 | 15.59 | [S25] |
| Al–Ti_3_C_2_T_x_/ANF  40/60 | 8.05 | 9.96 | [S26] |
| Ti_3_C_2_T_x_ /BC | 11.2 | 8.1 | [S27] |
| 2AMMO | 32.19 | 36.54 | This  Work |
| 4AMMO | 26.95 | 37.6 |  |
| 5AMMO | 25.78 | 26.27 |  |
| 6AMMO | 28.08 | 14.51 |  |
| 7AMMO | 24.47 | 9.44 |  |

**Table S4** EMI SE of ternary MXene/ANF-MoS_2_ composite films

|  | **Thickness(**$\boldsymbol{\mu}$**m)** | **SE_R_ (dB)** | **SE_A_ (dB)** | **SE_T_ (dB)** |
| --- | --- | --- | --- | --- |
| 2AMMO | 19 | 7.10 | 11.32 | 18.42 |
| 4AMMO | 20 | 13.23 | 12.64 | 25.87 |
| 5AMMO | 25 | 14.33 | 26.62 | 40.95 |
| 6AMMO | 33 | 13.88 | 30.00 | 43.88 |
| 7AMMO | 48 | 14.95 | 30.05 | 45.00 |

**Table S5** The comparison statistic between ternary MXene/ANF-MoS_2_ composite films and binary MXene/ANF composite films

| Material | SE_A_/SE_R_ | Strain-to-failure (%) | Toughness  (MJ∙m^-3^) | Electric heating  Temperature ($℃$) | Photothermal  conversion temperature ($℃$) | Reference |
| --- | --- | --- | --- | --- | --- | --- |
| 6AM | 1.54 | 18.80 | 8.82 | 88.6 | 43.8 | [S1] |
| 6AMMO | 2.16 | 28.08 | 14.51 | 91.6 | 53.7 | This work |

**Table S6** The comparison statistic between ternary MXene/ANF-MoS_2_ composite films and binary MXene/ANF composite films

| Material | SE_A_/SE_R_ | Strain-to-failure (%) | Toughness  (MJ∙m^-3^) | Tensile  strength ($℃$)) | SE_T_ ($℃$) | Reference |
| --- | --- | --- | --- | --- | --- | --- |
| 5AM | 1.49 | 21.86 | 12.83 | 104.65 | 40.02 | [1] |
| 5AMMO | 1.86 | 25.78 | 26.27 | 167.30 | 40.95 | This work |

**Table S7** Comparison statistic about filler content, strain-to-failure and EMI SE of the MXene/ANF composite films with other reported materials.

| **Material** | **Thickness(**$\boldsymbol{\mu}$**m)** | **Fequen-cy Range(GHz)** | **Filler content (wt%)** | **Strain-to-failure (%)** | **EMI SE**  **(dB)** | **Toughness**  **(MJ**$\boldsymbol{\cdot}$**m^-3^)** | **Refer-ences** |
| --- | --- | --- | --- | --- | --- | --- | --- |
| Ti_3_C_2_T_X_/BC | 6.7 | 8.2-12.4 | 44.9 | 7.6 | 25 | - | [S28] |
|  | 5.2 |  | 55.8 | 6.6 | 27 | - |  |
|  | 4 |  | 76.9 | 2.4 | 38 | - |  |
| Ti_3_C_2_T_X_/PEDOT:PSS | 15.2 | 8.2-12.4 | 75.0 | 1.5 | 9 | - | [S29] |
|  | 12.5 |  | 80.0 | 0.82 | 24 | - |  |
| Ti_3_C_2_T_X_/PEDOT:PSS | 6.6 | 8.2-12.5  and  11.9-18 | 70 | 0.3 | 42 | - | [S30] |
| Ti_3_C_2_T_X_/CNF | 167 | 8.2-12.4 | 50 | 16.7 | 25 | 14.8 | [S16] |
|  | 74 |  | 80 | 4.9 | 26 | 2.0 |  |
|  | 47 |  | 90 | 3.1 | 24 | 1.2 |  |
| Ti_3_C_2_T_X_/CNF | 35 | 8.2-12.4 | 52.8 | 4.3 | 41 | - | [S31] |
| Ti_3_C_2_T_X_/PVA | - | 8-12 | 35.5 | 5.0 | 37 | - | [S32] |
| Ti_3_C_2_T_X_/TOCNF | 33 | 8.2-12.4 | 30 | 3.6 | 29 | 4.1 | [S33] |
|  | 38 |  | 50 | 2.1 | 35 | 1.7 |  |
| PIF/ Ti_3_C_2_T_X_ | - | 8.2-12.4 | 13.8 | 10 | 22 | - | [S34] |
|  | 256 |  | 49.1 | 3 | 40 | - |  |
| Ti_3_C_2_T_X_ /cellulose | - | 8.2-12.4 | 20.0 | 4 | 34.9 | - | [S35] |
| PVDF/Ti_3_C_2_T_X_/AgNWs | 300 | 8.2-12.4 | 15 | 9.47 | 25 | - | [S36] |
| Ti_3_C_2_T_X_ /BCNFs | 11.4 | 8.2-12.4 | 50 | 2.6 | 42 | - | [S37] |
|  | - |  | 87.5 | 1.4 | 54 | - |  |
| Ti_3_C_2_T_X_ /SA/Ca^2+^ | 2.8 | 0.3-18 | 80 | 3.8 | 46 | - | [S38] |
| MXene/BC/APP | - | 8.2-12.4 | 80 | 4.3 | 41 | 2.9 | [S39] |
| MXene/ heterocyclic aramid | 16 | 8.2-12.4 | 50 | 6.9 | 25.9 | 16.29 | [S24] |
|  | 14.4 |  | 60 | 3.51 | 34.2 | 5.81 |  |
| Al–Ti_3_C_2_T_x_/ANF | - | 8.2-12.4 | 40 | 8.05 | 39.1 | 9.96 | [S26] |
| Ti_3_C_2_T_x_ /BC | - | 8.2-12.4 | 50 | 11.2 | 43.7 | 8.1 | [S27] |
| f-Ti_3_C_2_T_x_ /PNFs | 1705 | 8.2-12.4 | 50 | 16 | 28 | - | [S40] |
| MXene/MMT-CNF | - | 8.2-12.4 | 30 | 7 | 45 | - | [S41] |
| MXeneAgNW-CNF | 26 | 8.2-12.4 | 30 | 5.5 | 44 | - | [S42] |
| Ti_3_C_2_T_X_ /CMC | 3 | 0.3-18 | 90 | 4.5 | 53 | - | [S43] |
| Ti_3_C_2_T_X_ /ANF | 5.1 | 8.2-12.4 | 20 | 7 | 13 | 11 | [S44] |
|  | 4.5 |  | 40 | 2.8 | 28 | 4.5 |  |
|  | 3.9 |  | 60 | 1.5 | 33 | 2 |  |
|  | 3.2 |  | 80 | 1.7 | 39 | 1.8 |  |
| Ti_3_C_2_T_X_ /ANF | 23 | 8.2-12.4 | 20 | 8.45 | 12.74 | - | [S45] |
|  | 22 |  | 40 | 7.23 | 19.43 | - |  |
|  | 20 |  | 60 | 2.20 | 28.54 | - |  |
|  | 17 |  | 80 | 1.80 | 30.0 | - |  |
| Ti_3_C_2_T_X_ /ANF | - | 8.2-12.4 | 20 | 3.3 | 8 | 5.1 | [S46] |
|  | - |  | 40 | 3.0 | 26 | 5.3 |  |
|  | - |  | 60 | 1.74 | 35 | 1.2 |  |
|  | - |  | 80 | 1.75 | 42 | 1.1 |  |
| Ti_3_C_2_T_X_ /ANF | 12 | 8.2-12.4 | 10 | 13.3 | 27 | 13.7 | [S47] |
|  | 12 |  | 20 | 11.9 | 30 | 9.5 |  |
|  | 9 |  | 30 | 12.4 | 33 | 9.9 |  |
|  | 9 |  | 40 | 8.1 | 41 | 6.3 |  |
| Ti_3_C_2_T_X_ /ANF | - | 8.2-12.4 | 20 | 15.30 | 8 | 21.87 | [S22] |
|  | - |  | 40 | 7.52 | 19 | 13.39 |  |
|  | 40 |  | 60 | 5.41 | 39 | 6.01 |  |
|  | 37 |  | 80 | 3.26 | 50 | 2.37 |  |
| Ti_3_C_2_T_X_ /ANF | 10 | 8.2-12.4 | 20 | 12.45 | 24.17 | 19.97 | [S23] |
|  | 12 |  | 40 | 7.17 | 35.09 | 17.35 |  |
|  | 15 |  | 60 | 5.25 | 46.89 | 7.71 |  |
|  | 28 |  | 80 | 5.03 | 53.48 | 5.77 |  |
| Pure MXene | 3.4 | 0.3-18 | 100 | 2.26 | 60.9 | - | [S43] |
| This work | **19** | **8.2-12.4** | **40.42** | **32.19** | **18.42** | **36.54** |  |
|  | **20** |  | **52.28** | **26.95** | **25.87** | **37.6** |  |
|  | **33** |  | **65.85** | **28.08** | **43.88** | **14.51** |  |
|  | **48** |  | **73.41** | **24.47** | **45.00** | **9.44** |  |

**Supplementary References**

1. J. Wang, T. Song, W. Ming, M. Yele, L. Chen et al., High MXene loading, nacre-inspired MXene/ANF electromagnetic interference shielding composite films with ultralong strain-to-failure and excellent joule heating performance. Nano Res. **17**, 2061-2069 (2024). <https://doi.org/10.1007/s12274-023-6232-y>
2. P. Podsiadlo, A. K. Kaushik, E. M. Arruda, A. M. Waas, B. S. Shim et al., Ultrastrong and stiff layered polymer nanocomposites. Science **318**, 80-83 (2007). <https://doi.org/10.1126/science.1143176>
3. A. Walther, I. Bjurhager, J.-M. Malho, J. Pere, J. Ruokolainen et al., Large-area, lightweight and thick biomimetic composites with superior material properties via fast, economic, and green pathways. Nano Lett. **10**, 2742-2748 (2010). <https://doi.org/10.1021/nl1003224>
4. H.-B. Yao, Z.-H. Tan, H.-Y. Fang, S.-H. Yu, Artificial nacre-like bionanocomposite films from the self-assembly of chitosan–montmorillonite hybrid building blocks. Angew. Chem. Int. Ed. **49**, 10127-10131 (2010). <https://doi.org/10.1002/anie.201004748>
5. A. Walther, I. Bjurhager, J.-M. Malho, J. Ruokolainen, L. Berglund et al., Supramolecular control of stiffness and strength in lightweight high-performance nacre-mimetic paper with fire-shielding properties. Angew. Chem. Int. Ed. **49**, 6448-6453 (2010). <https://doi.org/10.1002/anie.201001577>
6. P. Das, S. Schipmann, J.-M. Malho, B. Zhu, U. Klemradt et al., Facile access to large-scale, self-assembled, nacre-inspired, high-performance materials with tunable nanoscale periodicities. ACS Appl. Mater. Interfaces **5**, 3738-3747 (2013). <https://doi.org/10.1021/am400350q>
7. S. Wan, J. Peng, Y. Li, H. Hu, L. Jiang et al., Use of synergistic interactions to fabricate strong, tough, and conductive artificial nacre based on graphene oxide and chitosan. ACS Nano **9**, 9830-9836 (2015). <https://doi.org/10.1021/acsnano.5b02902>
8. S. Wan, Y. Li, J. Peng, H. Hu, Q. Cheng et al., Synergistic toughening of graphene oxide–molybdenum disulfide–thermoplastic polyurethane ternary artificial nacre. ACS Nano **9**, 708-714 (2015). <https://doi.org/10.1021/nn506148w>
9. Y. Cheng, J. Peng, H. Xu, Q. Cheng, Glycera-inspired synergistic interfacial interactions for constructing ultrastrong graphene-based nanocomposites. Adv. Funct. Mater. **28**, 1800924 (2018). <https://doi.org/10.1002/adfm.201800924>
10. S. Gong, Q. Zhang, R. Wang, L. Jiang, Q. Cheng, Synergistically toughening nacre-like graphene nanocomposites via gel-film transformation. J. Mater. Chem. A **5**, 16386-16392 (2017). <https://doi.org/10.1039/C7TA03535G>
11. N. Zhao, M. Yang, Q. Zhao, W. Gao, T. Xie et al., Superstretchable nacre-mimetic graphene/poly(vinyl alcohol) composite film based on interfacial architectural engineering. ACS Nano **11**, 4777-4784 (2017). <https://doi.org/10.1021/acsnano.7b01089>
12. H. Zhao, Y. Yue, Y. Zhang, L. Li, L. Guo, Ternary artificial nacre reinforced by ultrathin amorphous alumina with exceptional mechanical properties. Adv. Mater. **28**, 2037-2042 (2016). <https://doi.org/10.1002/adma.201505511>
13. S. Wan, H. Hu, J. Peng, Y. Li, Y. Fan et al., Nacre-inspired integrated strong and tough reduced graphene oxide–poly(acrylic acid) nanocomposites. Nanoscale **8**, 5649-5656 (2016). <https://doi.org/10.1039/C6NR00562D>
14. A. Liu, A. Walther, O. Ikkala, L. Belova, L. A. Berglund, Clay nanopaper with tough cellulose nanofiber matrix for fire retardancy and gas barrier functions. Biomacromolecules **12**, 633-641 (2011). <https://doi.org/10.1021/bm101296z>
15. H. Sehaqui, A. Liu, Q. Zhou, L. A. Berglund, Fast preparation procedure for large, flat cellulose and cellulose/inorganic nanopaper structures. Biomacromolecules **11**, 2195-2198 (2010). <https://doi.org/10.1021/bm100490s>
16. W.-T. Cao, F.-F. Chen, Y.-J. Zhu, Y.-G. Zhang, Y.-Y. Jiang et al., Binary strengthening and toughening of MXene/cellulose nanofiber composite paper with nacre-inspired structure and superior electromagnetic interference shielding properties. ACS Nano **12**, 4583-4593 (2018). <https://doi.org/10.1021/acsnano.8b00997>
17. Y. Liu, S.-H. Yu, L. Bergström, Transparent and flexible nacre-like hybrid films of aminoclays and carboxylated cellulose nanofibrils. Adv. Funct. Mater. **28**, 1703277 (2018). <https://doi.org/10.1002/adfm.201703277>
18. Y. Wen, M. Wu, M. Zhang, C. Li, G. Shi, Topological design of ultrastrong and highly conductive graphene films. Adv. Mater. **29**, 1702831 (2017). <https://doi.org/10.1002/adma.201702831>
19. S. Gong, W. Cui, Q. Zhang, A. Cao, L. Jiang et al., Integrated ternary bioinspired nanocomposites via synergistic toughening of reduced graphene oxide and double-walled carbon nanotubes. ACS Nano **9**, 11568-11573 (2015). <https://doi.org/10.1021/acsnano.5b05252>
20. H. Li, X. Dai, X. Han, J. Wang, Molecular orientation-regulated bioinspired multilayer composites with largely enhanced mechanical properties. ACS Appl. Mater. Interfaces **15**, 21467-21475 (2023). <https://doi.org/10.1021/acsami.3c01647>
21. Y. Liu, N. Zhao, J. Xu, Mechanically strong and flame-retardant PBO/BN/MXene nanocomposite paper with low thermal expansion coefficient, for efficient EMI shielding and heat dissipation. Adv. Fiber Mater. **5**, 1657-1670 (2023). <https://doi.org/10.1007/s42765-023-00298-0>
22. J. Wang, X. Ma, J. Zhou, F. Du, C. Teng, Bioinspired, high-strength, and flexible MXene/aramid fiber for electromagnetic interference shielding papers with joule heating performance. ACS Nano **16**, 6700-6711 (2022). <https://doi.org/10.1021/acsnano.2c01323>
23. C. Liu, Y. Ma, Y. Xie, J. Zou, H. Wu et al., Enhanced electromagnetic shielding and thermal management properties in MXene/aramid nanofiber films fabricated by intermittent filtration. ACS Appl. Mater. Interfaces **15**, 4516-4526 (2023). <https://doi.org/10.1021/acsami.2c20101>
24. J. Xiong, R. Ding, Z. Liu, H. Zheng, P. Li et al., High-strength, super-tough, and durable nacre-inspired MXene/heterocyclic aramid nanocomposite films for electromagnetic interference shielding and thermal management. Chem. Eng. J. **474**, 145972 (2023). <https://doi.org/10.1016/j.cej.2023.145972>
25. F. Jia, J. Dong, X. Dai, Y. Liu, H. Wang et al., Robust, flexible, and stable CuNWs/MXene/ANFs hybrid film constructed by structural assemble strategy for efficient EMI shielding. Chem. Eng. J. **452**, 139395 (2023). <https://doi.org/10.1016/j.cej.2022.139395>
26. J. Wang, R. Zhai, X. Ma, W. Liu, C. Teng, Large flakes of Al–Ti_3_C_2_t_x_ MXene constructing highly ordered layered MXene/ANF films with integrated multifunctionalities. Ceram. Int. **50**, 11379-11391 (2024). <https://doi.org/10.1016/j.ceramint.2024.01.038>
27. H. Liu, Z. Cui, L. Luo, Q. Liao, R. Xiong et al., Facile fabrication of flexible and ultrathin self-assembled Ti_3_C_2_t*_x_*/bacterial cellulose composite films with multifunctional electromagnetic shielding and photothermal conversion performances. Chem. Eng. J. **454**, 140288 (2023). <https://doi.org/10.1016/j.cej.2022.140288>
28. Y. Wan, P. Xiong, J. Liu, F. Feng, X. Xun et al., Ultrathin, strong, and highly flexible Ti_3_C_2_t*_x_* MXene/bacterial cellulose composite films for high-performance electromagnetic interference shielding. ACS Nano **15**, 8439-8449 (2021). <https://doi.org/10.1021/acsnano.0c10666>
29. R. Liu, M. Miao, Y. Li, J. Zhang, S. Cao et al., Ultrathin biomimetic polymeric Ti_3_C_2_t*_x_* MXene composite films for electromagnetic interference shielding. ACS Appl. Mater. Interfaces **10**, 44787-44795 (2018). <https://doi.org/10.1021/acsami.8b18347>
30. Y.-J. Wan, X.-M. Li, P.-L. Zhu, R. Sun, C.-P. Wong et al., Lightweight, flexible MXene/polymer film with simultaneously excellent mechanical property and high-performance electromagnetic interference shielding. Composites, Part A **130**, 105764 (2020). <https://doi.org/10.1016/j.compositesa.2020.105764>
31. B. Zhou, Z. Zhang, Y. Li, G. Han, Y. Feng et al., Flexible, robust, and multifunctional electromagnetic interference shielding film with alternating cellulose nanofiber and MXene layers. ACS Appl. Mater. Interfaces **12**, 4895-4905 (2020). <https://doi.org/10.1021/acsami.9b19768>
32. W. Wang, A. C. Y. Yuen, H. Long, W. Yang, A. Li et al., Random nano-structuring of PVA/MXene membranes for outstanding flammability resistance and electromagnetic interference shielding performances. Composites, Part B **224**, 109174 (2021). <https://doi.org/10.1016/j.compositesb.2021.109174>
33. Z. Zhan, Q. Song, Z. Zhou, C. Lu, Ultrastrong and conductive MXene/cellulose nanofiber films enhanced by hierarchical nano-architecture and interfacial interaction for flexible electromagnetic interference shielding. J. Mater. Chem. C **7**, 9820-9829 (2019). <https://doi.org/10.1039/C9TC03309B>
34. K. Sun, F. Wang, W. Yang, H. Liu, C. Pan et al., Flexible conductive polyimide fiber/MXene composite film for electromagnetic interference shielding and joule heating with excellent harsh environment tolerance. ACS Appl. Mater. Interfaces **13**, 50368-50380 (2021). <https://doi.org/10.1021/acsami.1c15467>
35. M. Zhu, X. Yan, H. Xu, Y. Xu, L. Kong, Highly conductive and flexible bilayered MXene/cellulose paper sheet for efficient electromagnetic interference shielding applications. Ceram. Int. **47**, 17234-17244 (2021). <https://doi.org/10.1016/j.ceramint.2021.03.034>
36. H. Cheng, Y. Pan, Q. Chen, R. Che, G. Zheng et al., Ultrathin flexible poly(vinylidene fluoride)/MXene/silver nanowire film with outstanding specific emi shielding and high heat dissipation. Adv. Compos. Hybrid Mater. **4**, 505-513 (2021). <https://doi.org/10.1007/s42114-021-00224-1>
37. X. Xu, S. Wu, J. Cui, L. Yang, D. Liu et al., Insights into the microstructures and reinforcement mechanism of nano-fibrillated cellulose/MXene based electromagnetic interference shielding film. Cellulose **28**, 3311-3325 (2021). <https://doi.org/10.1007/s10570-021-03765-2>
38. S. Wan, X. Li, Y. Wang, Y. Chen, X. Xie et al., Strong sequentially bridged MXene sheets. Proc. Natl. Acad. Sci. U.S.A. **117**, 27154-27161 (2020). <https://doi.org/10.1073/pnas.2009432117>
39. S. Luo, Q. Li, Y. Xue, B. Zhou, Y. Feng et al., Reinforcing and toughening bacterial cellulose/MXene films assisted by interfacial multiple cross-linking for electromagnetic interference shielding and photothermal response. J. Colloid Interface Sci. **652**, 1645-1652 (2023). <https://doi.org/10.1016/j.jcis.2023.08.177>
40. L. Wang, Z. Ma, Y. Zhang, H. Qiu, K. Ruan et al., Mechanically strong and folding-endurance Ti_3_C_2_t*_x_* MXene/PBO nanofiber films for efficient electromagnetic interference shielding and thermal management. Carbon Energy **4**, 200-210 (2022). <https://doi.org/10.1002/cey2.174>
41. R. Cheng, Y. Wu, B. Wang, J. Zeng, J. Li et al., Fireproof ultrastrong all-natural cellulose nanofiber/montmorillonite-supported mxene nanocomposites with electromagnetic interference shielding and thermal management multifunctional applications. J. Mater. Chem. A **11**, 18323-18335 (2023). <https://doi.org/10.1039/D3TA03798C>
42. R. Cheng, B. Wang, J. Zeng, J. Li, J. Xu et al., Janus-inspired flexible cellulose nanofiber-assisted MXene/silver nanowire papers with fascinating mechanical properties for efficient electromagnetic interference shielding. Carbon **202**, 314-324 (2023). <https://doi.org/10.1016/j.carbon.2022.10.079>
43. S. Wan, X. Li, Y. Chen, N. Liu, Y. Du et al., High-strength scalable MXene films through bridging-induced densification. Science **374**, 96-99 (2021). <https://doi.org/10.1126/science.abg2026>
44. C. Weng, T. Xing, H. Jin, G. Wang, Z. Dai et al., Mechanically robust ANF/MXene composite films with tunable electromagnetic interference shielding performance. Composites, Part A **135**, 105927 (2020). <https://doi.org/10.1016/j.compositesa.2020.105927>
45. F. Xie, F. Jia, L. Zhuo, Z. Lu, L. Si et al., Ultrathin MXene/aramid nanofiber composite paper with excellent mechanical properties for efficient electromagnetic interference shielding. Nanoscale **11**, 23382-23391 (2019). <https://doi.org/10.1039/C9NR07331K>
46. C. Lei, Y. Zhang, D. Liu, K. Wu, Q. Fu, Metal-level robust, folding endurance, and highly temperature-stable MXene-based film with engineered aramid nanofiber for extreme-condition electromagnetic interference shielding applications. ACS Appl. Mater. Interfaces **12**, 26485-26495 (2020). <https://doi.org/10.1021/acsami.0c07387>
47. D. Hu, S. Wang, C. Zhang, P. Yi, P. Jiang et al., Ultrathin MXene-aramid nanofiber electromagnetic interference shielding films with tactile sensing ability withstanding harsh temperatures. Nano Res. **14**, 2837-2845 (2021). <https://doi.org/10.1007/s12274-021-3297-z>
